# Angiopoietin-like protein 3 governs LDL-cholesterol levels through endothelial lipase-dependent VLDL clearance

**DOI:** 10.1194/jlr.RA120000888

**Published:** 2020-07-09

**Authors:** Rene C. Adam, Ivory J. Mintah, Corey A. Alexa-Braun, Lisa M. Shihanian, Joseph S. Lee, Poulabi Banerjee, Sara C. Hamon, Hye In Kim, Jonathan C. Cohen, Helen H. Hobbs, Cristopher Van Hout, Jesper Gromada, Andrew J. Murphy, George D. Yancopoulos, Mark W. Sleeman, Viktoria Gusarova

**Affiliations:** 1 Regeneron Pharmaceuticals, Tarrytown, NY, USA; 2 Regeneron Genetics Center, Tarrytown, NY, USA; 3 Department of Molecular Genetics, University of Texas Southwestern Medical Center, Dallas, TX, USA; 4 Department of Internal Medicine, University of Texas Southwestern Medical Center, Dallas, TX, USA; 5 Howard Hughes Medical Institute, University of Texas Southwestern Medical Center, Dallas, TX, USA

**Keywords:** atherosclerosis, cardiovascular disease, familial hypercholesterolemia, low density lipoprotein-cholesterol, low density lipoprotein receptor, lipidomics, very low density lipoprotein

## Abstract

Angiopoietin-like protein (ANGPTL)3 regulates plasma lipids by inhibiting LPL and endothelial lipase (EL). ANGPTL3 inactivation lowers LDL-C independently of the classical LDLR-mediated pathway and represents a promising therapeutic approach for individuals with homozygous familial hypercholesterolemia due to *LDLR* mutations. Yet, how ANGPTL3 regulates LDL-C levels is unknown. Here, we demonstrate in hyperlipidemic humans and mice that ANGPTL3 controls VLDL catabolism upstream of LDL. Using kinetic, lipidomic, and biophysical studies, we show that ANGPTL3 inhibition reduces VLDL-lipid content and size, generating remnant particles that are efficiently removed from the circulation. This suggests that ANGPTL3 inhibition lowers LDL-C by limiting LDL particle production. Mechanistically, we discovered that EL is a key mediator of ANGPTL3’s novel pathway. Our experiments revealed that, although dispensable in the presence of LDLR, EL-mediated processing of VLDL becomes critical for LDLR-independent particle clearance. In the absence of EL and LDLR, ANGPTL3 inhibition perturbed VLDL catabolism, promoted accumulation of atypical remnants, and failed to reduce LDL-C. Taken together, we uncover ANGPTL3 at the helm of a novel EL-dependent pathway that lowers LDL-C in the absence of LDLR.

Hypercholesterolemia is a major risk factor for atherosclerosis and CAD (1). Despite advances in drug development designed to lower LDL-C levels, substantial risk of cardiovascular disease persists in patients with suboptimal responses to current medical interventions (2). Treatment options and efficacy of existing therapeutics remain even more limited for individuals with genetic mutations in the LDLR pathway [familial hypercholesterolemia (FH)]. Homozygous FH patients exhibit severely elevated LDL-C levels and develop CAD prematurely (3). While additional lipid-lowering strategies are required to achieve sufficient LDL-lowering in the general population, LDLR-independent pathways for patients lacking functional LDLR represent a particularly urgent need for therapeutic intervention.

Angiopoietin-like protein (ANGPTL)3 is an inhibitor of LPL and endothelial lipase (EL) and has emerged as a key regulator of vascular lipid metabolism (4). Individuals with *ANGPTL3* loss-of-function (LOF) alleles have low levels of plasma TGs, LDL-C, and HDL-C, and a decreased risk of CAD (5–9). Pharmacological inhibition of ANGPTL3 replicates the phenotype of individuals with *ANGPTL3* LOF mutations (6, 7) and lowers plasma lipids in mice, monkeys, and humans (9–11). Repression of vascular lipolysis by ANGPTL3 is central to the observed phenotype. While ANGPTL3 itself is a weak inhibitor of LPL, it also forms a codependent complex with ANGPTL8 for more robust inhibition of LPL. ANGPTL8 requires ANGPTL3 for secretion and unmasking of its potent LPL inhibitory motif (12, 13). Accordingly, inhibition of either ANGPTL3 or ANGPTL8 derepresses LPL and lowers plasma TG (11, 14–16). Furthermore, ANGPTL3 controls HDL-C levels through inhibition of EL (17). EL is a phospholipase that preferentially remodels HDL and promotes its clearance (18–22). Thus, ANGPTL3 inhibition derepresses EL and is associated with reduced plasma levels of HDL-C (11).

By contrast, the mechanism of how ANGPTL3 regulates LDL-C is unclear. Earlier studies revealed that ANGPTL3 blockade lowers LDL-C independently of LDLR (23, 24), providing a new therapeutic avenue for the treatment of FH (25). Previous attempts to explore the mechanism of LDL-C reduction with ANGPTL3 inhibition raised questions regarding the contribution of hepatic VLDL production. In mice, ANGPTL3 inhibition was found to be associated with reduced VLDL-TG secretion but had no effect on VLDL-APOB production (24). By contrast, human *ANGPTL3* LOF carriers were reported to have reduced VLDL-APOB production rates (6). Studies focused on lipoprotein clearance revealed that APOE or remnant receptors, LDLR-related protein 1 (LRP1) or syndecan-1 (SDC1), are not required for LDL-C reduction with ANGPTL3 blockade (24). Thus, it is still unclear whether ANGPTL3 inhibition lowers LDL-C by changing hepatic VLDL production, impacting LDL particle clearance in the circulation, or by modulating LDL-C production through upstream VLDL processing. Our current study aimed to address these questions in order to elucidate the ANGPTL3-driven novel LDLR-independent mechanism that regulates LDL-C levels.

Here, employing pharmacological, lipidomic, and kinetic studies in hyperlipidemic individuals and mice, we uncover an underappreciated role for EL beyond HDL catabolism. Specifically, we demonstrate that EL derepression by ANGPTL3 inhibition drives VLDL remodeling and clearance when LDLR is absent. This, in turn, depletes the LDL precursor pool, limits LDL particle production, and leads to reduction of plasma LDL-C levels. Our findings help to shape the conceptual view of how vascular lipase activity determines the fate of atherogenic lipoproteins and reveal alternative LDL-C-lowering mechanisms when the predominant pathway is defective.

## MATERIALS AND METHODS

### Mice and procedures

*Angptl3*^−/−^, *Ldlr*^−/−^, *Lipg*^−/−^, *Lipg*^−/−^*Ldlr*^−/−^, *Sdc1*^−/−^*Ldlr*^−/−^, and *Cd36*^−/−^ mice were generated by homologous recombination using VelociGene technology as described previously (26). *Apoe*^−/−^ mice were from JAX (#002052) (27). Male mice (one to five per cage) were maintained on a 12 h light/dark cycle at 22 ± 1°C and were fed ad libitum with chow (LabDiet, 5053). In some experiments, mice were fed a HFD (Research Diets, D12492; 60% fat by calories), as indicated in figure legends. Antibodies and antisense oligonucleotides (ASOs) were administered by subcutaneous injections. Mouse experiments were performed on age-matched and strain-matched pairs (littermates). All animal procedures were conducted in compliance with protocols approved by the Regeneron Pharmaceuticals Institutional Animal Care and Use Committee.

### Evinacumab clinical trial oversight and participants

The goal of the phase 1 first-in-human randomized single ascending dose placebo-controlled double-blind clinical study was to assess the safety, tolerability, and pharmacodynamics of REGN1500 (ANGPTL3 mAb/evinacumab) administered subcutaneously or intravenously to participants with varying degrees of dyslipidemia (28) (ClinicalTrials.gov identifier: NCT01749878). Part of the results for group A of this trial are described in this report. Group A enrolled healthy men and women 18 to 65 years of age with a fasting TG level of 150–450 mg/dl (1.7–5.1 mmol/l) or an LDL-C level of 100 mg/dl (2.6 mmol/l) or greater.

### Human genetic association analysis

The analysis was performed in subjects of European ancestry from the UK Biobank study with available exome sequences. Exome sequencing and variant calling were performed at the Regeneron Genetics Center as previously described (29). TG levels were log_10_-transformed. All traits were residualized, adjusting for age, age squared, sex, age-sex interaction, and 10 principal components of ancestry and subsequently transformed by rank-based inverse normal transformation. The association was tested using BOLT under linear mixed model (30). The effects in sd units and *P*-values were derived by testing the association against rank-based inverse normal transformed traits, while the effects in clinical units were derived by testing the association against residualized traits.

### Antibodies and ASOs

The fully human anti-ANGPTL3 (evinacumab, originally REGN1500) (11) mAb was derived using Regeneron’s VelocImmune technology platform (31, 32). An isotype-matched antibody with irrelevant specificity was used as control (REGN1945). Antibodies were diluted in saline and administered to mice (10 mg/kg for single dose, or 25 mg/kg for multi-dose) by subcutaneous injection. Evinacumab binds ANGPTL3 from multiple species, including mouse and human, with comparable affinity (11).

ASOs (Genscript USA Inc.) consisted of 2′-*O*-methoxyethyl-modified phosphorothioated DNA and 5-Me-dC nucleotides. ASOs were diluted in saline and administered to mice (25 mg/kg) twice per week by subcutaneous injection: control ASO (nontargeting), CCTTCCCTGAAGGTTCCTCC (33); *Lrp1* ASO, CCCAGTAGATGTTGCCTGCA (33); *Scarb1* ASO, GCTTCAGTCATGACTTCCTT (34).

### Single antibody administration studies

To establish a baseline for serum chemistry parameters, serum samples were collected 7 days prior to antibody administration in the nonfasted state (fed ad libitum). On study day 0, mice were sorted into treatment groups based on their serum LDL-C levels. Mice were administered single subcutaneous injections of REGN1500 or isotype control antibodies at the indicated doses. Subsequent serum samples were collected from nonfasted mice during the duration of the study and analyzed for serum chemistry parameters, FPLC or HPLC analyses, and APOB levels. Mice were euthanized 1 week after injection and livers were collected and frozen for subsequent lipid content and/or enzymatic measurements.

### Multiple antibody administration studies

Non-fasted baseline serum chemistry was determined on chow diet, and mice were sorted into treatment groups based on their LDL-C levels. Mice were then injected with REGN1500 or isotype control antibody (25 mg/kg sc) once a week for 3 weeks. Body weights were measured weekly. Mice were euthanized 1 week after the last injection and livers were collected and frozen for subsequent lipid content and/or enzymatic measurements or collected in RNA*later* (Thermo Fisher) for transcriptome studies. In some studies, mice were placed on HFD (Research Diets, D12492; 60% fat by calories) for 2 weeks; serum chemistry was measured again, and the mice were then sorted into treatment groups.

### Lipid analysis

Circulating TG, total cholesterol (TC), LDL-C, HDL-C, NEFA, alanine aminotransferase, and aspartate aminotransferase levels were determined in serum using an ADVIA® Chemistry XPT blood chemistry analyzer (Bayer). Non-HDL-C levels were calculated by subtracting HDL-C from TC values. Phospholipids were measured using a phospholipid C assay (Wako Diagnostics). Hepatic TBARS were measured using TBARS parameter assay kit (R&D) according to manufacturer’s instructions. Liver lipid levels were evaluated as previously described (11).

### Lipoprotein profiling

Lipoprotein particles were separated from serum samples by a dual detection HPLC system (Waters Corp.) using a tandem Superose 6 HR 10/300GL column. Cholesterol and TG lipoprotein concentrations were calculated as the area under the curve using VLDL, LDL, HDL (Biomedical Technologies), and TG (Teco Diagnostics) standards. The cholesterol or TG concentrations in the standards were determined using enzymatic methods (Teco Diagnostics). Alternatively, serum lipoprotein particles were size fractionated by FPLC using a Superose 6 Increase 10/300 GL column (GE Healthcare) and the cholesterol and TG content of each fraction was measured enzymatically (Infinity; Thermo Scientific).

### Lipoprotein clearance studies

VLDL and LDL were isolated by sequential ultracentrifugation from *Ldlr*^−/−^, *Lipg*^−/−^*Ldlr*^−/−^, or *Apoe*^−/−^ mice fed a chow diet and following a 4 h fast, and were then labeled in vitro with [^3^H]cholesteryl ether (CE) (Perkin Elmer). Equal volumes of either VLDL or LDL in saline were injected into the tail veins of *Ldlr*^−/−^ or *Apoe*^−/−^ mice. In some experiments, donor mice were treated with 2 weekly doses of ANGPTL3 mAb or isotype control mAb; while in other studies, recipient mice were mAb-treated as indicated. Recipient mice were fasted for 17 h, followed by 6 h of refeeding (ad libitum) before lipoprotein injections. Blood samples were collected at the indicated timepoints after the injection. Plasma was isolated from each sample and radioactivity was determined in 20 μl aliquots. The amount of [^3^H]CE lipoproteins remaining in the plasma was expressed as a percentage of the initial concentration, measured as the amount of [^3^H] radioactivity in the plasma at 30 s after injection. Lipoprotein half-life was then calculated by fitting an exponential to the decay curve plotted against time.

For clearance studies with [^125^I]LDL, LDL was isolated by sequential ultracentrifugation from *Ldlr*^−/−^ mice fed a chow diet and following a 4 h fast and were then labeled in vitro using the iodine monochloride method (Perkin Elmer). A total of 15 μg of [^125^I]LDL in saline was injected into the tail veins of *Ldlr*^−/−^ mice, which had previously been treated with 2 weekly doses of ANGPTL3 mAb or isotype control mAb. Plasma was isolated from each sample at the indicated timepoints, and radioactivity was measured by γ-counting (10 μl aliquot of plasma). A second 10 μl aliquot was precipitated with isopropanol, and the supernatant was counted. APOB labeling was calculated as the difference between total counts and the supernatant counts.

### Lipidomic analysis

#### Metabolite extraction and UHPLC-MS analysis.

Lipidomic analysis on separated lipoprotein fractions (VLDL, LDL, and HDL) was carried out by OWL Metabolomics (Derio, Spain). Metabolite extraction was accomplished by fractionating the samples into pools of species with similar physicochemical properties, using appropriate combinations of organic solvents. Two separate ultra-(U)HPLC-TOF-MS-based platforms that analyzed methanol and methanol/chloroform extracts were used to perform optimal profiling of lipid metabolites (35).

##### Platform 1.

Lipids analyzed in the methanol extract platform included fatty acids, oxidized fatty acids, bile acids, and lysoglycerophospholipids. Proteins were precipitated from 300 μl of the defrosted lipoprotein samples by adding 3 vol of methanol in 1.5 ml microtubes at room temperature. The methanol used for extraction was spiked with metabolites not detected in unspiked lipoprotein extracts. After brief vortex mixing, the samples were incubated for 1 h at −20°C. One thousand microliters of the supernatants were collected after centrifugation at 18,000 *g* for 5 min, dried, and reconstituted in 70 μl of methanol before being transferred to vials for UHPLC-MS analysis.

##### Platform 2.

The chloroform/methanol extract platform provided coverage over glycerolipids, cholesteryl esters (ChoEs), sphingolipids, and glycerophospholipids. Three volumes of chloroform/methanol (2:1) were added to 400 μl of defrosted lipoproteins after a brief vortex mixing. The chloroform/methanol used for extraction was spiked with metabolites not detected in unspiked lipoprotein extracts. After brief vortex mixing, the samples were incubated 1 h at −20°C. After centrifugation at 18,000 *g* for 5 min, 800 μl of the organic phase were collected and the solvent removed. Then, the extracts were dried and reconstituted in 100 μl of acetonitrile/isopropanol (1:1), centrifuged (18,000 *g* for 5 min), and transferred to vials for UHPLC-MS analysis. Specific chromatographic separation conditions and mass spectrometric detection conditions for each platform have been reported (35).

#### Quality controls.

Different types of quality control (QC) samples were used to assess the data quality. *1*) QC calibration samples were different extractions from a pool of all samples included in the study, used to correct the different response factors between and within batches. These samples were extracted and analyzed at the same time as the individual samples; *2*) QC Validation samples were reference serum samples, used to assess how well the data preprocessing procedure improved the data quality. As in the case of QC calibration samples, these samples were extracted and analyzed at the same time as the individual samples; *3*) QC blank samples were blank samples with extraction performed as for biological samples; and *4*) QC system suitability blank was a blank sample of the solvents in which biological samples were reconstituted. For each analytical platform, randomized sample injections were performed, with each of the QC calibration and validation extracts uniformly interspersed throughout the entire batch run.

#### Data preprocessing and normalization.

Following metabolite identification, all data were processed using the TargetLynx application manager for MassLynx 4.1 software (Waters Corp.). A set of predefined retention time mass-to-charge ratio pairs corresponding to metabolites included in the analysis was fed into the program. Associated extracted ion chromatograms (mass tolerance window = 0.05 Da) were then peak-detected and noise-reduced in both the LC and MS domains such that only true metabolite-related features were processed by the software. A list of chromatographic peak areas was then generated for each sample injection. An approximated linear detection range was defined for each identified metabolite, assuming similar detector response levels for all metabolites belonging to a given chemical class represented by a single standard compound (35). Metabolites for which more than 30% of data points were found outside their corresponding linear detection range were excluded from statistical analyses.

Normalization factors were calculated for each metabolite by dividing their intensities in each sample by the recorded intensity of an appropriate internal standard in that same sample, following the procedure described before (36).

#### Lipidomics data analysis.

Principal component analysis (PCA) was performed using SIMCA-P+ software package (version 14.1 Umetrics, Sweden), which showed that individual samples clustered together based on lipoprotein species (Fig. 2B). Univariate statistical analyses were also performed calculating group percentage changes and unpaired two-tailed Student’s *t*-test *P*-value (or Welch’s *t*-test where unequal variances were found) for the comparisons per lipoprotein (HDL, LDL, and VLDL). The Shapiro-Wilk test was used for testing the normality of data. After normality test, metabolites were evaluated by calculating group percentage changes and Student’s *t*-test *P*-value (normal distribution) or Wilcoxon rank-sum test (also called the Mann-Whitney U test) (non-normal distribution) for the unpaired comparisons included in the study.

These comparisons were used to generate heatmaps, which allow for visualization of the significant increments or decreases on the metabolic profile of a group of samples compared with another one. Green sections of the heatmap denote reduced metabolites (negative log_2_ fold-changes) and red sections denote metabolites increased (positive log_2_ fold-changes) in sample groups. Gray/black bars indicate significant *P*-values of the Student’s *t*-test (light gray, *P* < 0.05; dark gray, *P* < 0.01; black, *P* < 0.001). The metabolites are ordered by metabolic class, and in each class, individual features are ordered according to their carbon number and unsaturation degree of their acyl chains.

### Enzymatic assays

Microsomal TG transfer protein (MTP) and phospholipid transfer protein (PLTP) activity assays (Roar Biomedical, Inc.) were measured according to manufacturer’s instructions. Serum APOB levels were measured using Mouse APOB ELISA (Kamiya Biomedical).

### Western blot analysis

Samples were resolved by SDS-PAGE using Criterion™ TGX™ 4–20% precast gel (Bio-Rad) under reducing conditions and transferred to PVDF membranes (Immobilon P). The membranes were blocked with 5% milk in 0.1% Tween-20 and probed with the indicated antibodies and detected using an enhanced chemiluminescent detection system (Millipore). APOB antibody (Millipore, AB742) was used at 1:1,000. Purified mouse LDL was used as positive control (see full scan Western blot images in supplemental data).

### RNA preparation and RNA-sequencing read mapping

Total RNA was purified from *Ldlr*^−/−^ livers (n = 6 isotype controls, n = 5 ANGPTL3 mAb, following 3 weekly doses of 25 mg/kg) using MagMAX™-96 for Microarrays Total RNA Isolation kit (Ambion by Life Technologies), according to manufacturer’s specifications. Genomic DNA was removed using MagMAX™Turbo™DNase buffer and TURBO DNase from the MagMAX kit listed above (Ambion by Life Technologies). mRNA was purified from total RNA using Dynabeads® mRNA purification kit (Invitrogen). Strand-specific RNA sequencing (RNA-seq) libraries were prepared using KAPA mRNA-Seq Library Preparation kit (Kapa Biosystems). Twelve-cycle PCR was performed to amplify libraries. Sequencing was performed on Illumina HiSeq®2500 by a multiplexed single-read run with 33 cycles. Raw sequence data (BCL files) were converted to FASTQ format via Illumina bcl2fastq v2.17. Reads were decoded based on their barcodes and read quality was evaluated with FastQC (www.bioinformatics.babraham.ac.uk/projects/fastqc/). Reads were mapped to the mouse genome (NCBI GRCm38) using ArrayStudio® software (OmicSoft®, Cary, NC) allowing two mismatches. Reads mapped to the exons of a gene were summed at the gene level. Differentially expressed genes were identified by DESeq2 (37) package and significantly perturbed genes were defined with fold changes of no less than 1.5 in either up or down direction and with *P*-values of at least 0.01.

### Statistical analysis

Statistical and graphical data analyses were performed using Microsoft Excel and Prism 7 (GraphPad Software, Inc.). Data are expressed as mean ± SEM. Mean values were compared using unpaired two-tailed *t*-tests, one-way or two-way ANOVA as implemented in the GraphPad Prism 7.0 software (GraphPad Software, Inc.). In box and whisker plots, the middle line is plotted at the median, the upper and lower hinges correspond to the first and third quartiles, and the upper and lower whiskers display the full range of variation (minimum to maximum). Grubbs’ test was used to determine and remove significant outliers.

## RESULTS

### EL is necessary for the LDLR-independent LDL-C-lowering effect of ANGPTL3 inhibition

**Fig. 1.**
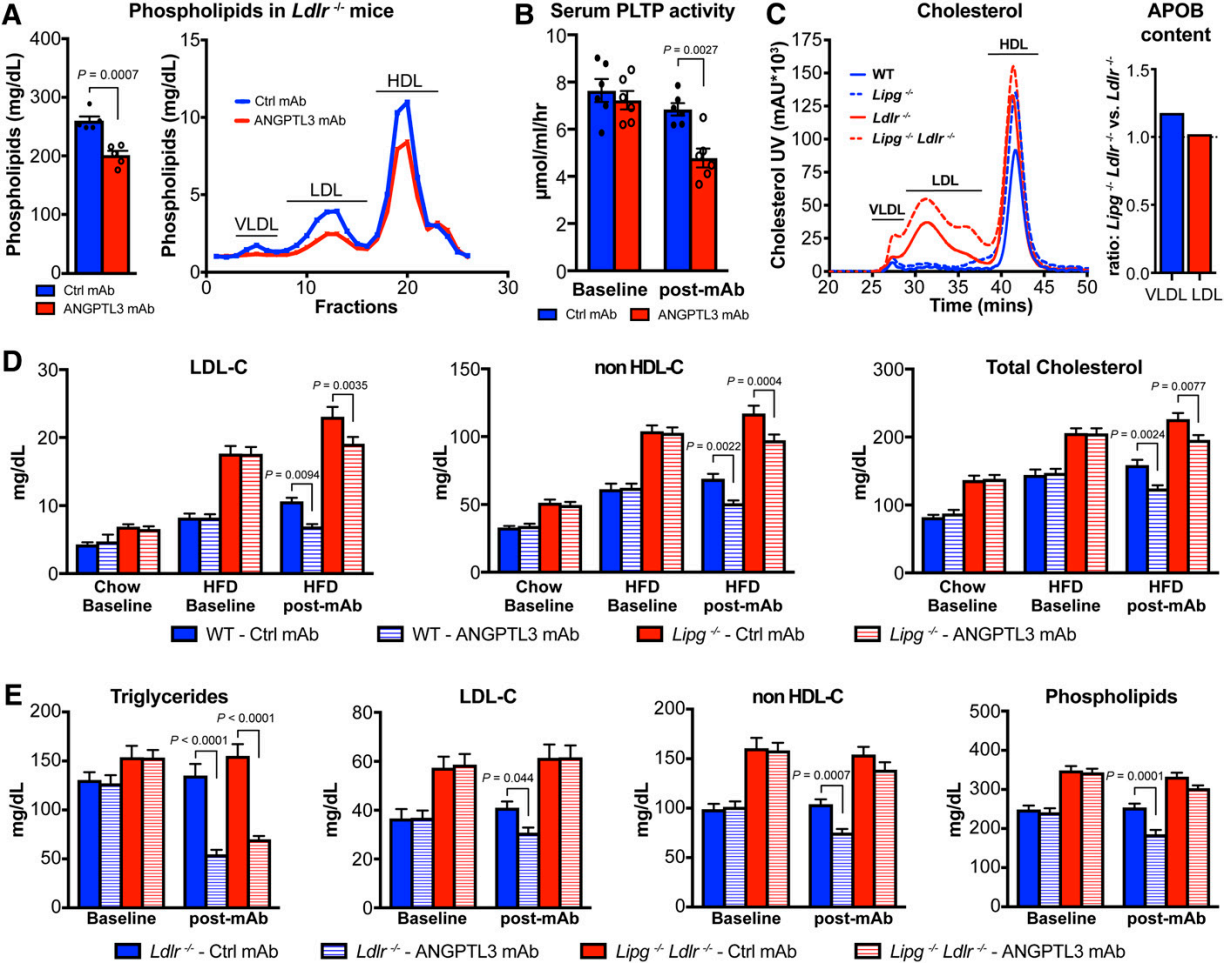
EL is necessary for the LDLR-independent LDL-C-lowering effect upon ANGPTL3 inhibition. A: Inhibition of ANGPTL3 lowers serum phospholipids in *Ldlr*^−/−^ mice on chow diet 4 days after ANGPTL3 mAb or control mAb (n = 5 mice per group, 10 mg/kg mAb) (left). Mean ± SEM are shown. *P*-values are from unpaired two-tailed Student’s *t*-test. Serum lipid distribution in *Ldlr*^−/−^ mice showing ANGPTL3 mAb lowers phospholipids across all lipoproteins. Pooled serum [n = 6 mice in control (Ctrl) mAb group; n = 5 mice in ANGPTL3 mAb group] was fractionated by FPLC, and phospholipid levels were measured enzymatically in each fraction (right). B: Serum PLTP activity in *Ldlr*^−/−^ mice on chow diet 6 days before (baseline) and 7 days after ANGPTL3 mAb or control mAb (n = 6 mice per group, 10 mg/kg mAb). Mean ± SEM are shown. *P*-values are from two-way ANOVA with Sidak correction posttest relative to control. C: Serum lipid distribution of chow-fed WT (n = 10), *Lipg*^−/−^ (n = 12), *Ldlr*^−/−^ (n = 23), and *Lipg*^−/−^*Ldlr*^−/−^ (n = 24) mice (left). Pooled serum from each group of mice was separated by HPLC, and cholesterol levels were measured enzymatically. Ratio showing VLDL and LDL APOB-particle numbers in *Ldlr*^−/−^ and *Lipg*^−/−^*Ldlr*^−/−^ mice (right). D: Nonfasted serum cholesterol of WT and *Lipg*^−/−^ mice on chow (baseline), after 2 weeks on HFD (HFD baseline), and 6 days after ANGPTL3 or control mAb dose (25 mg/kg) while on HFD. Mean ± SEM are shown (n = 15 mice in WT Ctrl mAb group, n = 16 mice in all other groups). *P*-values are from two-way ANOVA with Tukey correction posttest. E: Nonfasted serum lipids of *Ldlr*^−/−^ and *Lipg*^−/−^*Ldlr*^−/−^ mice on chow diet before (baseline, day −6) and 7 days after ANGPTL3 or control mAb-dose (10 mg/kg). Mean ± SEM are shown (n = 14 mice in each *Ldlr*^−/−^ group, n = 17 mice in *Lipg*^−/−^*Ldlr*^−/−^ Ctrl mAb group, n = 18 mice in *Lipg*^−/−^*Ldlr*^−/−^ ANGPTL3 mAb group). *P*-values are from two-way ANOVA with Sidak correction posttest.

To investigate the LDLR-independent mechanism of LDL-C reduction, we employed monoclonal ANGPTL3 antibody (evinacumab/REGN1500) (11) to inactivate circulating ANGPTL3 in *Ldlr*-deficient mice. Consistent with previous findings, ANGPTL3 antibody lowered plasma levels of TG and cholesterol independently of LDLR (24) (supplemental Fig. S1A, B). Additional analysis revealed that ANGPTL3 inhibition also lowered serum phospholipid levels (**Fig. 1A**), indicative of derepression of EL, whose phospholipase activity is responsible for HDL-C reduction upon ANGPTL3 inhibition (11). FPLC analysis provided insights into the composition of individual lipoproteins and showed that beyond HDL, ANGPTL3 inhibition also led to reduction in VLDL- and LDL-phospholipids (Fig. 1A, supplemental Fig. S1B). In order to understand whether the reduction in phospholipids is driven by their transfer from VLDL to HDL as a consequence of LPL-hydrolysis (38), we evaluated the activity of the responsible enzyme, PLTP (39). We found that evinacumab lowered PLTP activity, making it unlikely to explain the reduction in VLDL-phospholipids (Fig. 1B). Instead, the data suggested that ANGPTL3 inhibition promotes phospholipid hydrolysis on VLDL. While LPL has low phospholipase activity in vitro, both EL and hepatic lipase (HL) are the predominant phospholipases in vivo [reviewed in (20)]. However, we have previously shown that HL is unaffected by ANGPTL3 (11, 40). Therefore, we hypothesized that ANGPTL3 inhibition may enable EL to impact APOB-containing lipoproteins and contribute to the LDLR-independent LDL-C-lowering effect.

To test our hypothesis, we employed EL-deficient mice (*Lipg*^−/−^), which exhibit elevated HDL-C and circulating phospholipids on chow diet (19) (see HPLC in Fig. 1C, supplemental Fig. S2A). While *Lipg*^−/−^ mice also showed a trend toward increased LDL-C levels (supplemental Fig. S2A), these effects were more difficult to assess, as mice typically have very low LDL-C on chow diet. We therefore subjected *Lipg*^−/−^ mice to HFD (60% kcal from fat) to elevate baseline LDL-C levels before administering evinacumab. Curiously, ANGPTL3 inhibition reduced LDL-C and TC to a similar extent in WT and *Lipg*^−/−^ mice (Fig. 1D), suggesting either that EL is entirely dispensable for LDL-C reduction upon ANGPTL3 inhibition, or the availability of LDLR allows for clearance of atherogenic particles in *Lipg*^−/−^ mice and hence masks the role of EL.

To answer this question, we generated mice deficient in both EL and LDLR (*Lipg*^−/−^*Ldlr*^−/−^). The combined deletion of *Lipg* and *Ldlr* exacerbated the lipid phenotypes on chow diet, with *Lipg*^−/−^*Ldlr*^−/−^ mice displaying substantially higher cholesterol levels than *Ldlr*^−/−^ mice (Fig. 1C, supplemental Fig. S2A) (41). In addition to further elevated HDL-C, the increase in VLDL-C and LDL-C was particularly notable (Fig. 1C). *Lipg*^−/−^*Ldlr*^−/−^ mice also displayed increased APOB levels (present as single copy per VLDL and LDL particle) in comparison to *Ldlr*^−/−^ mice (supplemental Fig. S2A). Further analysis revealed that the APOB content of LDL was similar and APOB content of VLDL was increased by ∼20% in *Lipg*^−/−^*Ldlr*^−/−^ compared with *Ldlr*^−/−^ mice (Fig. 1C). The greatly raised cholesterol content in *Lipg*^−/−^*Ldlr*^−/−^ mice (Fig. 1C, supplemental Fig. S2A) contrasted with the minor elevation in particle numbers (judged by APOB content), suggesting that APOB-lipoproteins carried more lipids per particle when EL and LDLR were absent.

Next, we tested the effect of ANGPTL3 inhibition on lipid levels in these mice on chow diet. Administration of evinacumab led to TG reduction in both *Ldlr*^−/−^ and *Lipg*^−/−^*Ldlr*^−/−^ mice (Fig. 1E). However, in contrast to EL single ablation, double EL/LDLR KO rendered evinacumab ineffective in reducing LDL-C, non-HDL-C, and phospholipids (Fig. 1E). The HPLC analysis further revealed the differential effect of ANGPTL3 inhibition on all cholesterol fractions between *Ldlr*^−/−^ and *Lipg*^−/−^*Ldlr*^−/−^ mice, demonstrating that EL is necessary for evinacumab-driven LDLR-independent LDL-C reduction (supplemental Fig. S2B). Taken together, our studies identified EL as key mediator of a novel LDL-C-lowering pathway operating in the absence of LDLR.

To evaluate whether EL contributes to LDL-C metabolism in humans, we queried the UK Biobank for genetic associations of known *LIPG* variants with serum lipids (up to 180,374 individuals, supplemental Table S1). The *LIPG* Asn396Ser variant (MAF = 0.0134), which severely blunts EL enzymatic activity, was associated with increased HDL-C (effect: +4.25 mg/dl, *P* = 1.7 × 10^−101^), as previously reported (42). More modest but still significant increases in plasma levels of LDL-C (effect: +2.1 mg/dl, *P* = 7.3 × 10^−6^) and non-HDL-C (effect: +2.85 mg/dl, *P* = 2.8 × 10^−6^) were observed in association with this missense variant. In contrast, the common *LIPG* Thr111Ile variant (MAF = 0.299) that does not alter EL activity (42) was not associated with changes in LDL-C (effect: +0.21 mg/dl, *P* = 5.7 × 10^−2^) and non-HDL-C (effect: +0.26 mg/dl, *P* = 6.9 × 10^−2^). Although carriers of both *LDLR* and *EL* LOF alleles have not been identified to date, the human genetic data are consistent with our mouse studies showing that EL contributes to APOB-lipoprotein metabolism, beyond its established role in HDL remodeling.

### Lipidomic analysis reveals the impact of EL on LDL composition

**Fig. 2.**
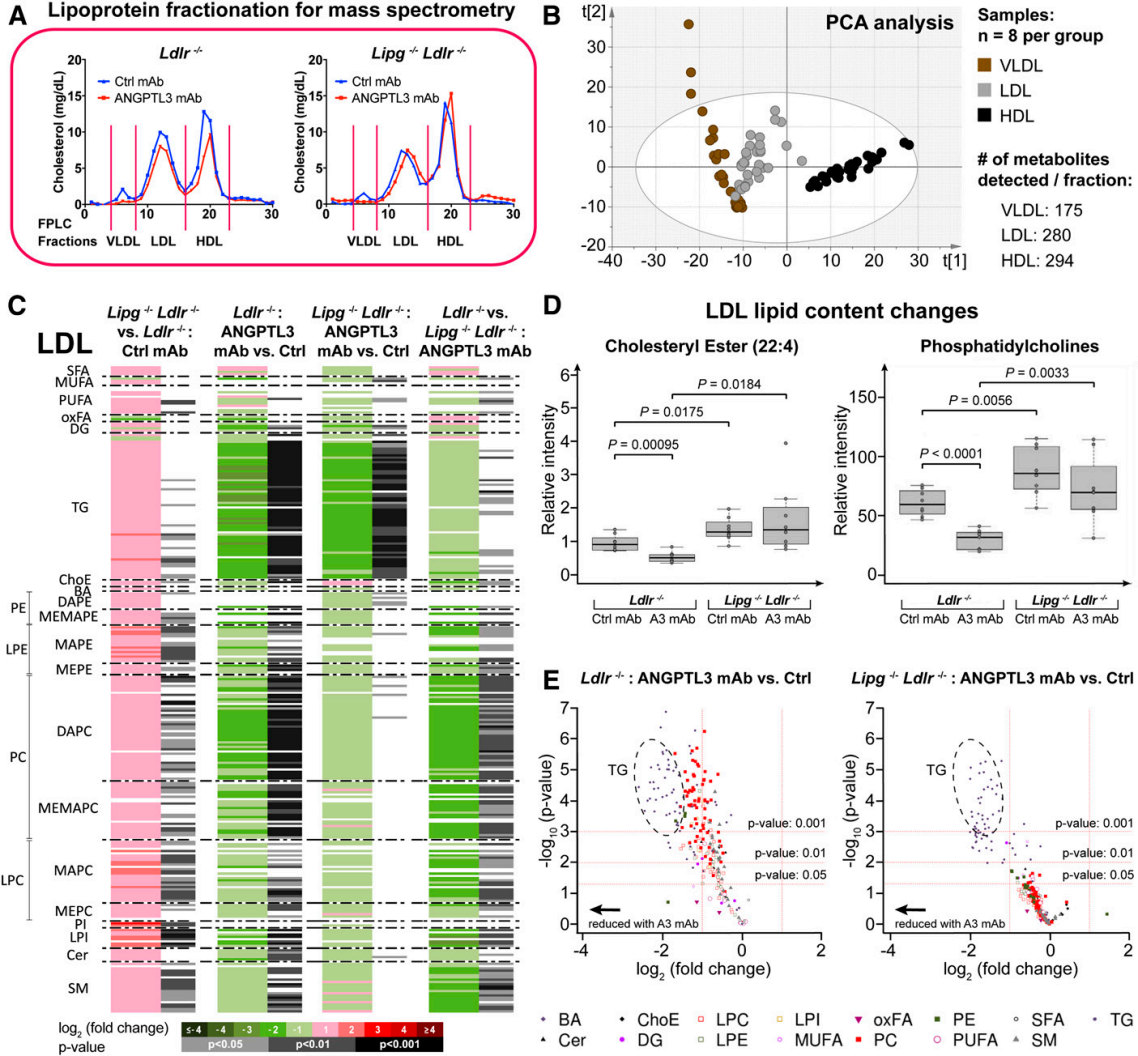
Lipidomic analysis reveals the impact of EL on LDL composition. A: Sample preparation for lipidomic analysis. Serum of chow-fed *Ldlr*^−/−^ and *Lipg*^−/−^*Ldlr*^−/−^ individual mice was collected 7 days after mAb administration and subjected to FPLC analysis to separate lipoproteins. Fractions corresponding to VLDL/LDL/HDL were pooled and snap-frozen for subsequent metabolite extraction. B: Score scatter plot (unsupervised PCA) showing clustering of lipidomics samples depending on lipoprotein species. Model diagnostics: A = 6; R2X = 0.872; Q2X = 0.813. C: Heatmap representing individual metabolites (denoted on the left side) obtained for the comparisons performed in LDL samples (n = 8 mice per group). Heatmap color codes for log_2_ (fold-change) and unpaired two-tailed Student’s *t*-test *P*-values are indicated at the bottom. D: Boxplots (defined in the Materials and Methods) of LDL-lipids upon ANGPTL3 mAb or isotype control. *P*-values are from unpaired two-tailed Student’s *t*-test. E: Volcano plots showing impact of ANGPTL3 inhibition on LDL in *Ldlr*^−/−^ and *Lipg*^−/−^*Ldlr*^−/−^ mice. All mice were on chow diet. See also lipidomics data in supplemental Table S3.

Mechanistically, it is not well-understood how increased EL activity impacts APOB-containing lipoprotein homeostasis. To address this question, we evaluated how ANGPTL3 blockade alters lipoprotein composition in the presence or absence of EL by employing a MS-based lipidomic approach. We utilized VLDL, LDL, and HDL fractions obtained by FPLC separation of serum from chow-fed *Ldlr*^−/−^ and *Lipg*^−/−^*Ldlr*^−/−^ mice treated with either an isotype control antibody or evinacumab (eight biological replicates per group, **Fig. 2A**). To maximize metabolite recovery from the lipoproteins, two extraction methods (methanol or chloroform/methanol) were used prior to performing UHPLC-MS (see Materials and Methods).

The proximity among QC injections provided a good indication of the reproducibility of the measurements (supplemental Fig. S3A). Unsupervised PCA showed that individual samples clustered based on lipoprotein species (Fig. 2B). The number of distinctly detected metabolites comprised 175 in VLDL, 280 in LDL, and 294 in HDL (Fig. 2B). As expected, VLDL was rich in TG, while LDL exhibited an abundance of ChoEs and lipolytic products, including lysophospholipids (lysophosphatidylcholine, lysophosphatidylyethanolamine, lysophosphatidylinositol), diglycerides, and ceramides (supplemental Fig. S3A). HDL contained the greatest and most diverse number of lipids, including large amounts of PCs, PEs, sphingomyelins, and polyunsaturated fatty acids.

Analyses of HDL from *Lipg*^−/−^*Ldlr*^−/−^ mice revealed an enrichment of phospholipids, especially PCs and PIs, as well as ChoE (supplemental Fig. S3B, first column; and supplemental Table S2). Prior in vitro studies had shown that PI is a preferred substrate of EL (43). More broadly, our findings corroborated EL’s relevance to HDL-phospholipid catabolism: ANGPTL3 inhibition led to reduction of HDL-phospholipids in *Ldlr*^−/−^ mice but had little effect in *Lipg*^−/−^*Ldlr*^−/−^ mice (supplemental Fig. S3B, two middle columns; supplemental Fig. S3C). Conversely, HDL-TG reduction appeared to be driven by LPL derepression, as TGs were similarly reduced in *Ldlr*^−/−^ and *Lipg*^−/−^*Ldlr*^−/−^ mice (supplemental Fig. S3C). Although HDL was not our main focus, the data supporting a requirement for EL in HDL-phospholipid, but not TG-turnover following ANGPTL3 inhibition, helped to validate our approach.

Intriguingly, the effects of EL ablation on LDL lipid composition and amount were as striking as those seen with HDL (Fig. 2C, first column). Despite equivalent LDL particle numbers between *Ldlr*^−/−^ and *Lipg*^−/−^*Ldlr*^−/−^ mice (based on APOB content of LDL fraction, Fig. 1C), LDL from *Lipg*^−/−^*Ldlr*^−/−^ mice comprised increased contents of ChoE and glycerophospholipids. Several TG species were also significantly enriched, albeit to a lesser extent (Fig. 2C, first column). Conversely, ANGPTL3 inhibition in *Ldlr*^−/−^ mice reversed this pattern and yielded significant reductions in >85% of individual phospholipid and TG species (Fig. 2C, second column; supplemental Table S3). EL played a critical role in mediating these effects: whereas TG-hydrolysis was similarly effective in *Ldlr*^−/−^ and *Lipg*^−/−^*Ldlr*^−/−^ mice, EL-deficiency almost abrogated evinacumab’s effect on LDL-phospholipid and ChoE content. Although many glycerophospholipid species trended lower, few reached statistical significance (Fig. 2C, third column; Fig. 2D). Hence without EL, ANGPTL3 inhibition led to the formation of atypical TG-depleted phospholipid/ChoE-rich LDL particles (Fig. 2C, third column; Fig. 2E).

### EL promotes VLDL catabolism and APOB particle reduction

**Fig. 3.**
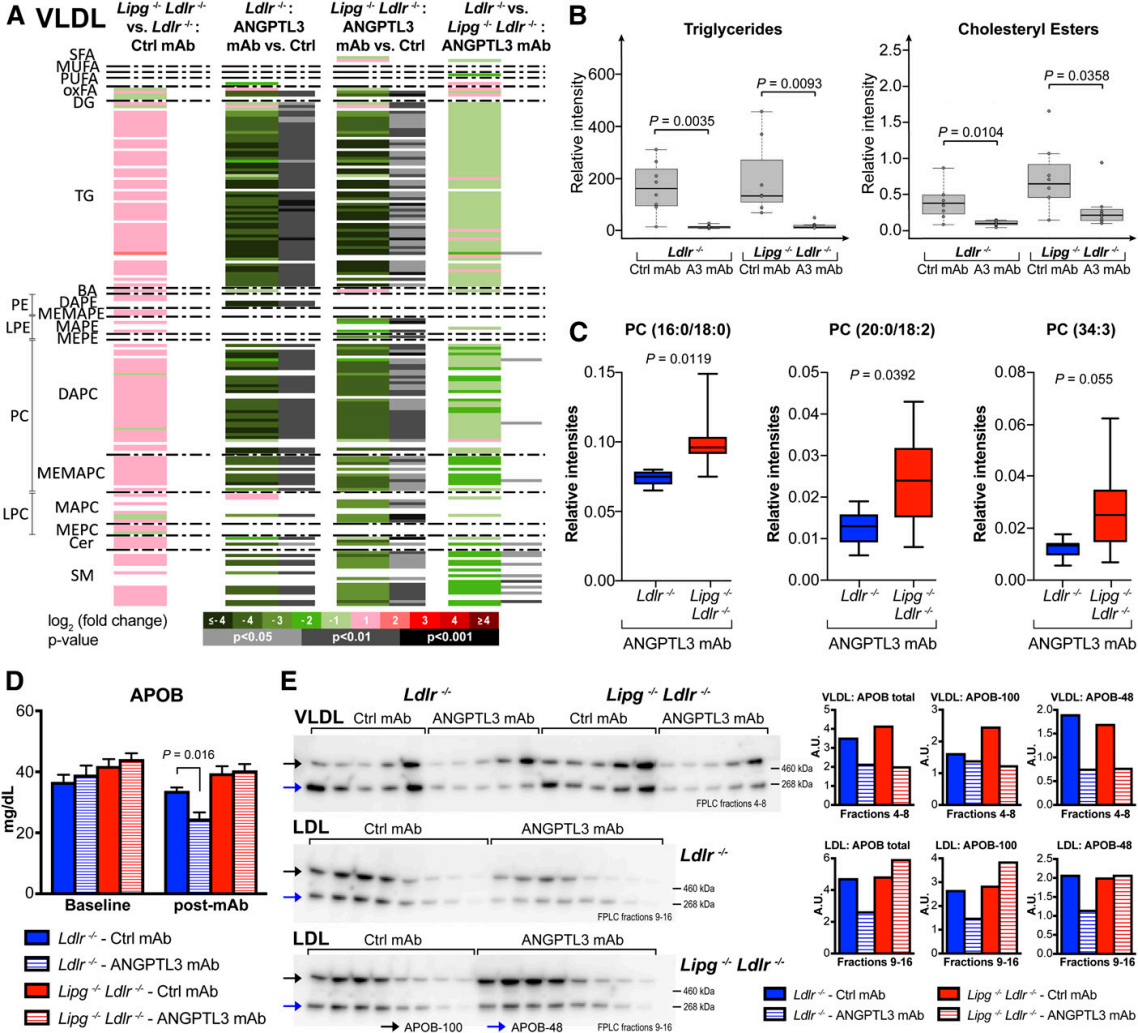
EL promotes VLDL catabolism and APOB particle reduction. A: Heatmap of lipidomics analysis, from the study outlined in Fig. 2, representing individual metabolites (denoted on the left side) obtained for the comparisons performed in VLDL samples (n = 8 mice per group). Heatmap color codes for log_2_ (fold-change) and unpaired two-tailed Student’s *t*-test *P*-values are indicated at the bottom. B: Boxplots (defined in the Materials and Methods) of VLDL-lipids upon ANGPTL3 mAb or control antibody treatment. *P*-values are from unpaired two-tailed Student’s *t*-test. C: Boxplots (defined in the Materials and Methods) of VLDL-PCs in *Ldlr*^−/−^ and *Lipg*^−/−^*Ldlr*^−/−^ mice upon ANGPTL3 inhibition. *P*-values are from unpaired two-tailed Student’s *t*-test. D: Serum APOB levels before (baseline) and 4 days after ANGPTL3 mAb administration. Mean ± SEM are shown (n = 14 mice in *Ldlr*^−/−^ groups, n = 18 mice in *Lipg*^−/−^*Ldlr*^−/−^ groups). *P*-value is from two-way ANOVA with Sidak correction posttest. E: APOB Western blot analysis. Serum of *Ldlr*^−/−^ (n = 7) and *Lipg*^−/−^*Ldlr*^−/−^ mice (n = 9) was collected 4 days after mAb administration, pooled, and lipoproteins were separated by FPLC. Fractions corresponding to VLDL/LDL were immunoblotted and probed with anti-APOB antibody. Densitometry quantifications are on the right. Samples were derived from the same experiment and gels/blots were processed in parallel. Full scans of Western blots are provided in the supplemental data online. All mice were on chow diet. See also lipidomics data in supplemental Table S4.

To determine whether the altered lipid content of LDL was caused by changes in its metabolic precursor, we examined the lipid composition of VLDL. The reduction in VLDL-lipid content was greater than that seen for LDL with ANGPTL3 inhibition (compare the two middle columns of **Fig. 3A** and Fig. 2C). Most remarkable was the dramatic reduction in VLDL-TG: ANGPTL3 inhibition led to a >10 times reduction of 73% (45/62) of VLDL-TG species in *Ldlr*^−/−^ mice. It similarly lowered 85% (44/52) of VLDL-TGs by >10 times in *Lipg*^−/−^*Ldlr*^−/−^ mice, indicative of unrestrained LPL activity (Fig. 3A, two middle columns; Fig. 3B; supplemental Table S4).

On the other hand, dissecting the role of EL derepression in VLDL-processing and cholesterol reduction turned out to be more complex than we initially envisioned. Unlike for LDL and HDL, evinacumab diminished VLDL-C to similar extents in *Ldlr*^−/−^ and *Lipg*^−/−^*Ldlr*^−/−^ mice; hence EL appeared dispensable for evinacumab-driven VLDL-phospholipid reduction (Fig. 3A, two middle columns; Fig. 3B). While at first glance these data suggested little contribution of EL to VLDL metabolism, closer inspection revealed that ANGPTL3 inhibition lowered VLDL-phospholipids by approximately two times more in LDLR- versus EL/LDLR-deficient mice. PCs were particularly affected, evident from reduced VLDL-PC species in *Ldlr*^−/−^ relative to *Lipg*^−/−^*Ldlr*^−/−^ mice (Fig. 3C, supplemental Table S4).

Given that VLDL is the precursor for LDL, the lipidomic data raised the question of how the extensively processed VLDL (TG^low^ PL^low^ ChoE^low^) could give rise to phospholipid/ChoE-enriched LDL (TG^low^ PL^High^ ChoE^high^) when EL is missing (Figs. 2C, E; 3A). To reconcile these results, we evaluated how ANGPTL3 inhibition affects the relative distribution of VLDL and LDL particles by using APOB levels as a proxy readout. It is noteworthy that APOB and LDL-C levels were similarly influenced by EL (Figs. 1E, 3D): while ANGPTL3 inhibition lowered the total number of APOB-containing particles in *Ldlr*^−/−^ mice (Fig. 3D), it had no effect on APOB levels in *Lipg*^−/−^*Ldlr*^−/−^ mice, mimicking the effect on LDL. To determine particle distribution upon ANGPTL3 inhibition, we performed APOB Western blots on FPLC-separated serum from chow-fed *Ldlr*^−/−^ and *Lipg*^−/−^*Ldlr*^−/−^ mice. In *Ldlr*^−/−^ mice, evinacumab reduced APOB in VLDL and LDL fractions by similar amounts, suggestive of particle clearance (Fig. 3E). In *Lipg*^−/−^*Ldlr*^−/−^ mice, ANGPTL3 inhibition lowered APOB in VLDL to the same extent as in *Ldlr*^−/−^ mice. However, it increased APOB in LDL fractions, indicative of particle accumulation. Hence, whereas total APOB was largely unchanged in the absence of EL, evinacumab triggered APOB-containing lipoprotein redistribution (Fig. 3D, E). The increased conversion of VLDL to LDL followed by LDL accumulation suggested generation of atypical remnants and defects in APOB-containing particle clearance when EL was missing. Indeed, the resulting particles represent the phospholipid/ChoE-rich LDL that we identified by MS (Fig. 2E).

Taken together, our data suggest that ANGPTL3 inhibition elicits APOB-containing lipoprotein catabolism and promotes EL-driven modifications to facilitate LDLR-independent particle uptake. Without EL/LDLR, partially processed LDL accumulates, and clearance is perturbed (Fig. 3E). That said, the data do not allow us to unequivocally determine which particles undergo clearance. ANGPTL3 inhibition, via derepression of EL, could either drive VLDL remnant uptake, removing LDL precursor, or it could directly promote LDL clearance to lower LDL-C.

### ANGPTL3 inhibition promotes VLDL processing and clearance

**Fig. 4.**
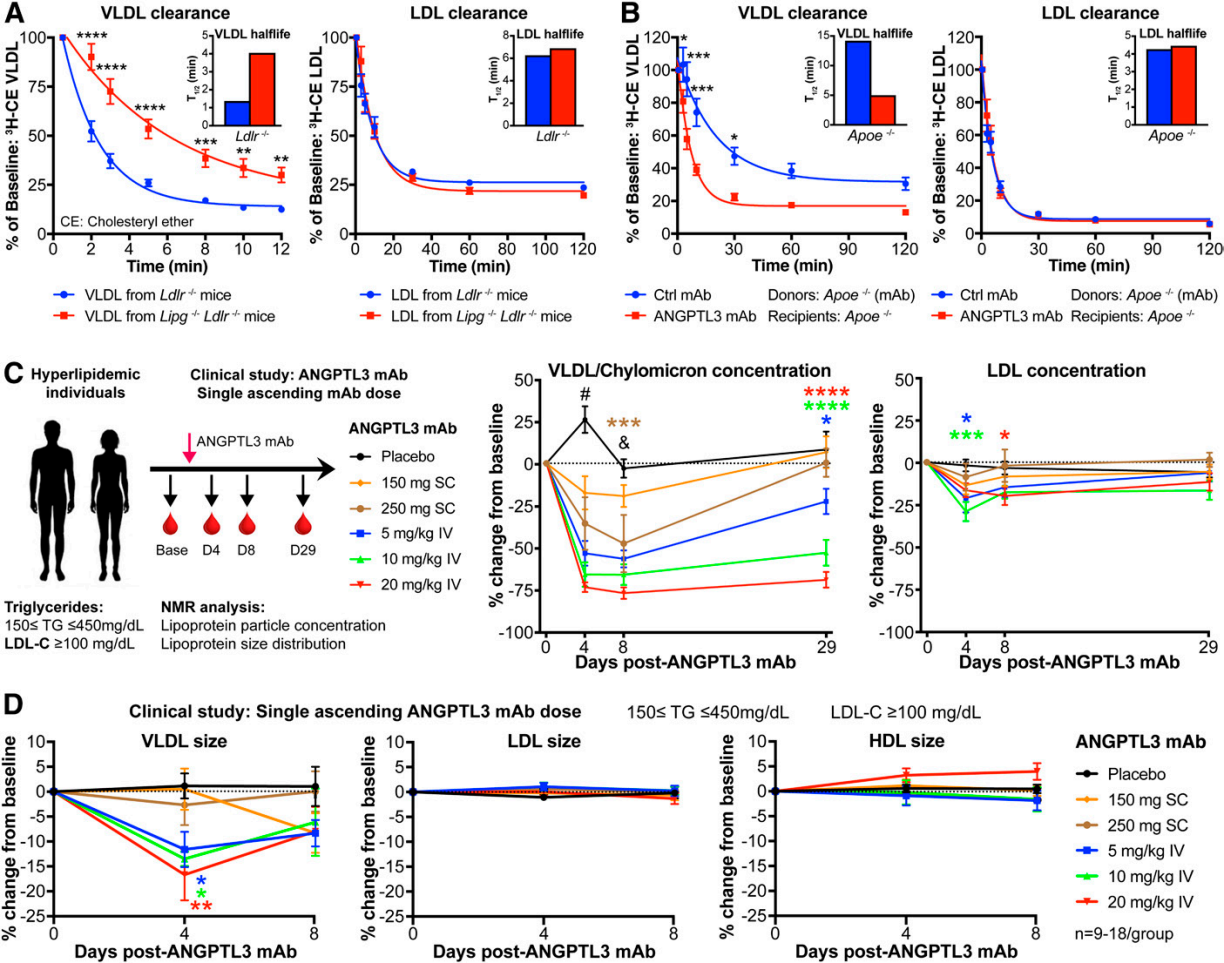
ANGPTL3 inhibition promotes VLDL processing and clearance. A: Plasma clearance kinetics of [^3^H]CE-labeled VLDL (left) and LDL (right) isolated from *Ldlr*^−/−^ or *Lipg*^−/−^*Ldlr*^−/−^ mice and injected into *Ldlr*^−/−^ mice (VLDL from *Ldlr*^−/−^: n = 10 recipient mice; VLDL from *Lipg*^−/−^*Ldlr*^−/−^: n = 9 recipient mice). B: Plasma clearance kinetics of [^3^H]CE-labeled VLDL (left) or LDL (right) isolated from *Apoe*^−/−^ mice after 2 weekly injections of ANGPTL3 mAb or control mAb and injected into *Apoe*^−/−^ mice (n = 10 mice per group). A, B: The percentage of injected CE-label remaining at each timepoint was determined using the value obtained at 30 s as starting point. Mean ± SEM are shown for each timepoint. *P*-values from two-way ANOVA with Sidak correction posttest: *P < 0.05, **P < 0.01, ***P < 0.001, ****P < 0.001. The half-life was calculated from the decay curve of [^3^H]CE activity plotted against time. All mice were on chow diet. C, D: NMR analysis of human lipoproteins following ANGPTL3 inhibition with evinacumab (ANGPTL3 mAb). Hyperlipidemic individuals were administered ANGPTL3 mAb or control mAb (phase 1 single ascending dose clinical study) (28), and serum was collected at the indicated timepoints. Mean ± SEM are shown for each timepoint (n = 18 in placebo; n = 10 in 5 mg/kg iv; n = 9 in 10 mg/kg iv; n = 11 in 20 mg/kg iv; n = 12 in 150 mg sc; and n = 9 in 250 mg sc groups). Human VLDL/chylomicron and LDL concentration (C) and particle size analysis by NMR (D). *P*-values are from two-way ANOVA with Tukey correction posttest: *P < 0.05, **P < 0.01, ***P < 0.001, ****P < 0.0001 relative to placebo (color of asterisks indicates relevant group); ^#^*P* < 0.0001 regarding all ANGPTL3 mAb groups relative to placebo; ^&^*P* < 0.0001 regarding all intravenously administered ANGPTL3 mAb groups relative to placebo.

To evaluate the relevance of EL-modifications on LDLR-independent lipoprotein uptake, we performed plasma clearance kinetic studies in *Ldlr*^−/−^ mice. VLDL and LDL were isolated from *Ldlr*^−/−^ or *Lipg*^−/−^*Ldlr*^−/−^ donor mice, radiolabeled with [^3^H]CE, and injected into *Ldlr*^−/−^ recipients to monitor their rates of disappearance from the circulation (**Fig. 4A**, left panel). Notably, clearance of VLDL was significantly delayed when donor mice lacked EL. In contrast, the absence of EL did not affect LDL kinetics (Fig. 4A, right panel).

Next, we assessed how ANGPTL3 influences LDLR-independent lipoprotein clearance. Neither LDL APOB-labeled particles ([^125^I]LDL) nor LDL-C ([^3^H]CE-LDL) clearance in *Ldlr*^−/−^ mice was altered by evinacumab (supplemental Fig. S4A, B). Evaluation of VLDL clearance proved more challenging, as its rapid half-life in WT or *Ldlr*^−/−^ mice (∼1.5 min) precluded current and prior studies from detecting changes in VLDL uptake (supplemental Fig. S4C) (24). To overcome this, we performed studies in mice deficient for APOE, the absence of which impedes normal VLDL uptake and afforded us a larger window to detect potential changes in evinacumab-driven VLDL clearance (44, 45). Furthermore, it was previously shown that *Apoe* deletion has no bearing on evinacumab’s lipid-lowering effect (24) (supplemental Fig. S4D) and would therefore not confound data interpretation. We injected [^3^H]CE-labeled VLDL or LDL, purified from evinacumab or isotype control-treated donor *Apoe*^−/−^ mice, into *Apoe*^−/−^ recipients and monitored plasma removal. Remarkably, evinacumab greatly accelerated VLDL clearance (Fig. 4B). Again, no differences were seen for LDL clearance in *Apoe*^−/−^ mice. Thus, the mouse data suggested that ANGPTL3 inhibition predominantly modifies VLDL catabolism.

To understand whether ANGPTL3 inhibition elicited similar effects on VLDL remodeling in humans, we performed lipoprotein analyses using samples of participants of the evinacumab clinical trial (28) (phase 1 single ascending dose study). Hyperlipidemic human individuals (LDL-C ≥100 mg/dl, 150 ≤ TG ≤ 450 mg/dl) received a single ascending dose of evinacumab or placebo by either subcutaneous or intravenous administration to evaluate the optimal dosing regimen (Fig. 4C). At the indicated timepoints, blood was collected and subjected to NMR analysis to determine lipoprotein characteristics. The magnitude and duration of lipid reductions were dose proportional, with evinacumab significantly reducing VLDL/chylomicron particles by 77% at the highest dose tested. The effect on LDL particle reduction was more modest (29%) and similar in magnitude to HDL (33%; Fig. 4C, supplemental Fig. S4E). Furthermore, NMR provided important insights into changes in lipoprotein size, which was not readily apparent from the mouse studies. Specifically, evinacumab reduced VLDL/chylomicron but not LDL or HDL particle size in humans (Fig. 4D). Thus, the clinical data suggested that evinacumab does not alter LDL remodeling directly and provided further evidence that ANGPTL3 inhibition primarily affects VLDL.

Taken together, our studies emphasized the importance of ANGPTL3/EL in governing VLDL remodeling in hyperlipidemic humans and mice. Whereas VLDL remnants could be generated without EL (Fig. 3), its VLDL phospholipid-modifications appeared to be necessary for LDLR-independent particle removal at the juncture between VLDL remnants and their processing to become LDL.

### ANGPTL3 has no impact on hepatic lipid homeostasis

The data above suggested that ANGPTL3 governs LDL-C levels by controlling vascular lipolysis and VLDL clearance. Prior studies have proposed additional, yet conflicting, roles for ANGPTL3 in regulating hepatic lipid metabolism. In mice, ANGPTL3 inhibition has been shown to lower liver lipid content (10) and to reduce hepatic VLDL-TG secretion without impacting VLDL-APOB production (24). By contrast, decreased VLDL-APOB production rates were reported in human individuals with *ANGPTL3* LOF variants (6). To further evaluate how ANGPTL3 regulates hepatic VLDL production to influence plasma LDL-C, we investigated VLDL assembly in LDLR-deficient mice in greater detail.

Liver transcriptome analysis of *Ldlr*^−/−^ mice revealed no impact of ANGPTL3 inhibition on hepatic mRNA expression, including genes related to APOB-lipidation or lipases EL (*Lipg*) and HL (*Lipc*) (supplemental Fig. S5A, supplemental Table S5). These findings were consistent with the data reported in *Angptl3*-deficient mice (24). Furthermore, posttranslational mechanisms of VLDL assembly (46) were unperturbed by evinacumab: neither MTP nor intracellular PLTP activities were affected, mirroring our findings in *Angptl3*^−/−^ mice (supplemental Fig. S5B). Lipid peroxidation, which at elevated levels is associated with presecretory VLDL degradation (46), was also unchanged (supplemental Fig. S5B). Evinacumab had no impact on liver lipids in *Ldlr*^−/−^ mice, even after multi-dose treatment, and no changes in liver fat were seen in *Angptl3*^−/−^ mice (supplemental Fig. S5C) (40). Collectively, our data show that ANGPTL3 has no apparent effect on hepatic VLDL assembly, although we cannot rule out secondary effects as a result of increased lipolysis and return of lipid substrates to the liver.

### Multiple remnant receptors could contribute to VLDL clearance upon ANGPTL3 inhibition

Given our new insights into the role of EL in APOE- and LDLR-independent VLDL clearance, we postulated that unrestrained EL may promote VLDL uptake via SR-B1, akin to its action on HDL (22, 47). To address this, we used ASOs for in vivo knockdown of chow-fed SR-B1/*Scarb1* in *Ldlr*^−/−^ mice (34). SR-B1 ASO yielded ∼90% *Scarb1* knockdown in liver and significantly elevated serum cholesterol (supplemental Fig. S6A–C). Despite a 3.5-fold increase in non-HDL-C over baseline, evinacumab potently lowered cholesterol in APOB-lipoproteins and hence did not require SR-B1 (supplemental Fig. S6A–C). Of note, ANGPTL3 inhibition had no effect on HDL-C in *Scarb1* ASO-treated mice, consistent with SR-B1’s established role as HDL-receptor (supplemental Fig. S6A–C) (22).

**Fig. 5.**
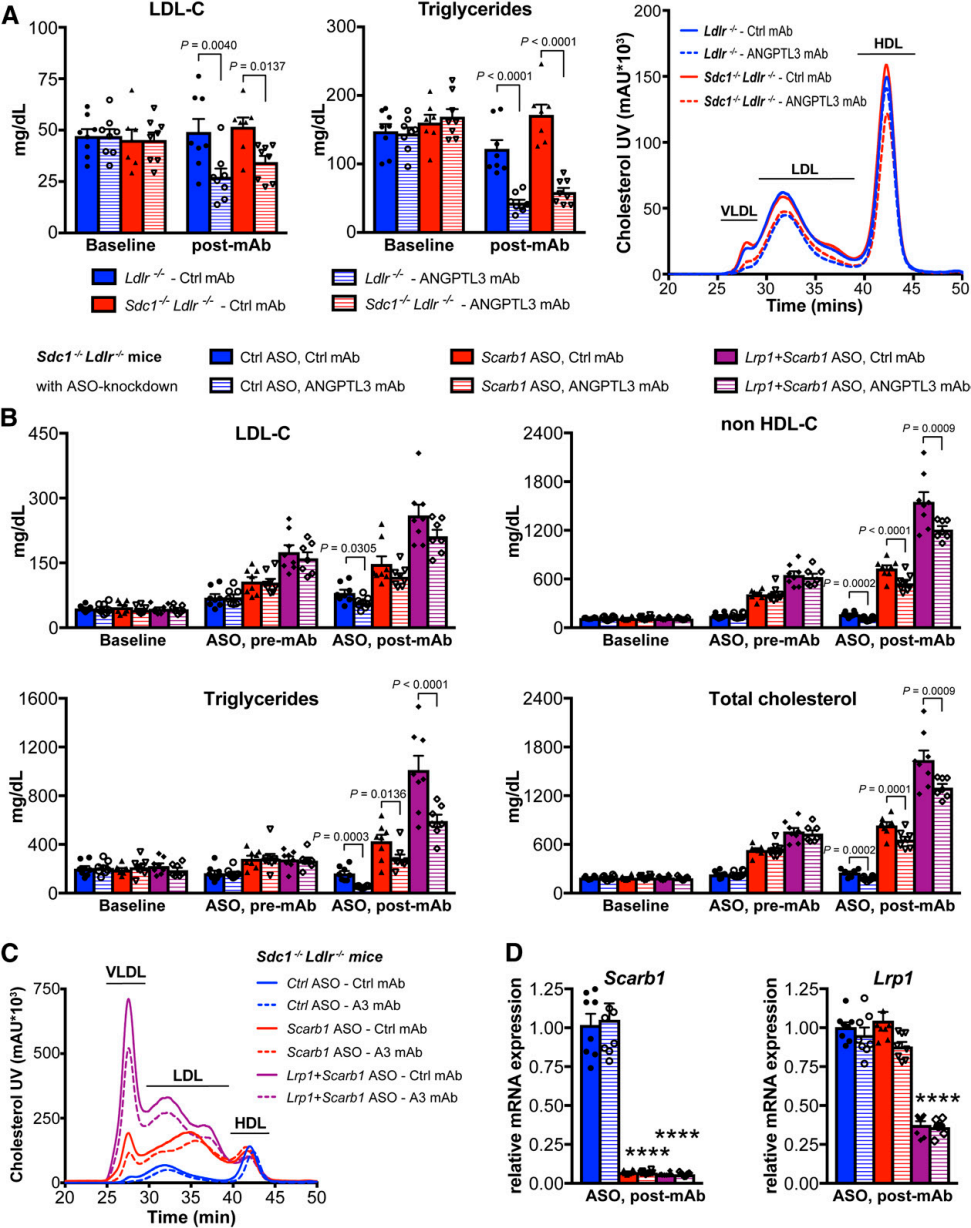
ANGPTL3 inhibition lowers LDL-C independently of several lipoprotein receptors. A: Nonfasted serum lipids of *Ldlr*^−/−^ and *Sdc1*^−/−^*Ldlr*^−/−^ mice on chow diet before (baseline, day −6) and 7 days after ANGPTL3 or control mAb-dose (10 mg/kg). Mean ± SEM are shown [n = 7 mice in *Sdc1*^−/−^*Ldlr*^−/−^ control (Ctrl) mAb group, n = 8 mice in all other groups]. *P*-values are from two-way ANOVA with Sidak correction posttest. HPLC analysis shown at right. B, C: The effect of ANGPTL3 inhibition on serum lipids in ASO-mediated knockdown of *Scarb1* and/or *Lrp1* in *Sdc1*^−/−^*Ldlr*^−/−^ mice on chow diet. Nonfasted serum was collected from *Sdc1*^−/−^*Ldlr*^−/−^ mice (baseline). *Scarb1*, *Scarb1+Lrp1*, or control ASOs (25 mg/kg) were administered twice weekly by subcutaneous injections, for a total of 10 doses. Two once-weekly doses of ANGPTL3 or control mAb (10 mg/kg) were administered after first six doses of ASO (ASO pre-mAb). Seven days after the second mAb dose, serum was collected and livers were harvested (ASO, post-mAb). Mean lipid levels ± SEM are shown in B; HPLC analysis of serum is shown in C (n = 8 mice in all groups except n = 7 in *Scarb1*+*Lrp1* ASO + ANGPTL3 mAb group). *P*-values are from two-way ANOVA with Sidak correction posttest. D: Hepatic knockdown efficiency upon ASO-mediated knockdown of *Scarb1* and/or *Lrp1* in *Sdc1*^−/−^*Ldlr*^−/−^ mice, corresponding to the study shown in B and C. Mean ± SEM are shown (n = 8 mice per group except n = 7 mice in *Scarb1*+*Lrp1* ASO + ANGPTL3 mAb group). *P*-values are from one-way ANOVA with Tukey correction posttest. ****P < 0.0001 for *Scarb1* ASO groups or *Lrp1+Scarb1* ASO groups relative to control ASO groups.

Probing further, we considered heparan sulfate proteoglycan SDC1, a low-affinity high-capacity remnant receptor capable of binding lipoproteins via EL (48, 49). Although evinacumab effectively lowered plasma cholesterol and TGs in *Sdc1*^−/−^ mice in earlier studies, the presence of functional LDLR may again have masked the effects by allowing for APOB-particle clearance (24). Hence, we generated mice deficient in both SDC1 and LDLR (*Sdc1*^−/−^*Ldlr*^−/−^). However, evinacumab reduced cholesterol and TG to similar extents in *Sdc1*^−/−^*Ldlr*^−/−^ and control mice (**Fig. 5A**). We found that ANGPTL3 inhibition did not rely on CD36 either (supplemental Fig. S7), and prior data excluded hepatic LRP1 as a candidate receptor (24).

Given the multitude of remnant receptors known to compensate for each other (47, 50, 51), we hypothesized that a combination of receptors could mediate VLDL remnant clearance upon ANGPTL3 inhibition. We therefore generated quadruple lipoprotein receptor-deficient mice by administering ASOs targeting SR-B1 alone or in combination with LRP1 ASO (33, 34) to *Sdc1*^−/−^*Ldlr*^−/−^ mice (Fig. 5B). Knockdown of additional receptors yielded a gene dosage-dependent increase in serum cholesterol: targeting SR-B1 strikingly elevated cholesterol (834 mg/dl), while combined SR-B1 and LRP1 ASO treatment further accentuated these effects in *Sdc1*^−/−^*Ldlr*^−/−^ mice on chow diet (1639 mg/dl vs. 252 mg/dl for Ctrl ASO). However, evinacumab still potently reduced serum cholesterol, even in mice with reduced or absent expression of four major VLDL remnant receptors (Fig. 5B, C).

Given the widespread expression of *Lrp1* and *Scarb1*, incomplete knockdown in the liver (Fig. 5D) and in extrahepatic tissues (data not shown) could provide an explanation for this effect. On the other hand, additional remnant receptors (50, 52, 53) could further compensate and mediate evinacumab-driven VLDL uptake. The potential for redundancy was already apparent from our studies in *Lipg* (single) KO mice with functional LDLR, in which evinacumab lowered LDL-C likely through LDLR (Fig. 1D). That said, we cannot completely rule out nonreceptor mediated clearance mechanisms, or other contributing factors, e.g., potential effects of EL on hepatic VLDL secretion. While intriguing, exploring these options is beyond the scope of the present investigation. Most importantly, the notion that ANGPTL3 inhibition appears to engage multiple pathways underscores the efficiency and versatility of this pathway, and provides an explanation for its potent reduction of LDL-C.

## DISCUSSION

**Fig. 6.**
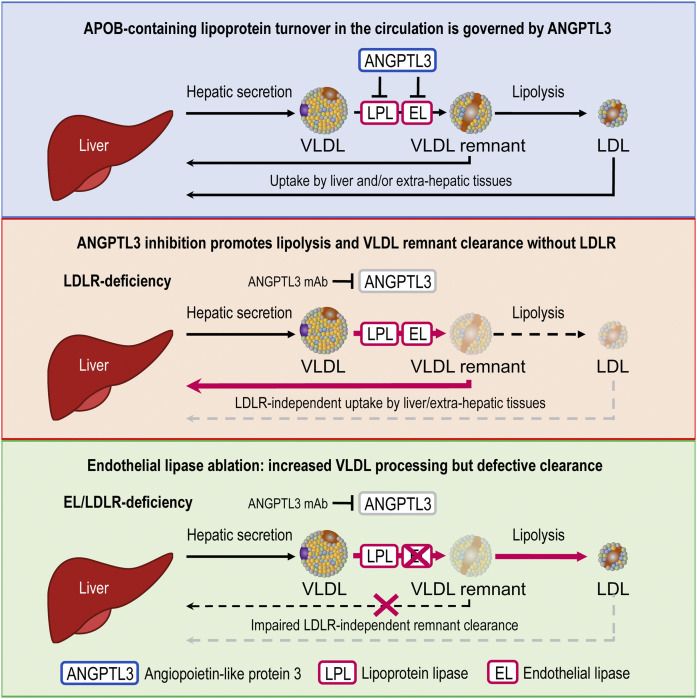
ANGPTL3 inhibition lowers LDL-C through VLDL remodeling and remnant clearance. Schematic depicting the mechanism of how ANGPTL3 inhibition lowers LDL-C. Upper panel: During homeostasis, ANGPTL3 curbs vascular lipases LPL and EL and thus regulates APOB-containing lipoprotein turnover. Middle panel: ANGPTL3 inhibition leads to derepression of both lipases, promoting VLDL remodeling and preferential removal of VLDL remnants from the circulation via redundant receptors. ANGPTL3 inhibition hence lowers LDL-C by limiting vascular LDL production. Lower panel: EL is critical in the process: without EL, LDLR-independent clearance mechanisms are perturbed, leading to accumulation of LDL particles and rendering ANGPTL3 inhibition ineffective in the reduction of LDL-C levels.

Epidemiological studies over the past decades have placed increasing emphasis on the importance of LDL-C reduction in the prevention of CAD (2, 54). Despite the availability of a number of lipid-lowering therapies, treatment options remain limited for patients with deficiencies in the LDLR pathway. ANGPTL3 inhibition has emerged as a promising strategy for these patients, as it is associated with a dramatic reduction of both cholesterol and TGs and lowers LDL-C independently of LDLR (9, 11, 24, 25). While the pathways contributing to TG and HDL-C reduction with ANGPTL3 inactivation have been well characterized (11, 14, 17), much less is known about the mechanism responsible for its LDL-C-lowering effect. Here, we reveal an under-appreciated role for EL in APOB-containing lipoprotein metabolism. Importantly, we demonstrate that ANGPTL3’s regulation of EL is not limited to its effect on HDL-C but is required for the modulation of LDL-C levels when LDLR is absent. Mechanistically, we show that EL derepression by evinacumab leads to extensive remodeling of VLDL, resulting in formation of lipid-depleted remnant particles, which accelerates their clearance from the circulation. This, in turn, leads to depletion of the LDL precursor pool and reduces LDL-C levels (**Fig. 6**). Such an EL-dependent alternative pathway likely functions unnoticed alongside LDLR during homeostasis, but becomes more relevant when LDLR is dysfunctional.

While a prior study using EL overexpression hinted at the potential role of EL in the catabolism of APOB-containing lipoproteins (55), *Lipg*^−/−^*Ldlr*^−/−^ mice proved instrumental in uncovering new concepts under physiologically relevant conditions. By employing lipidomic and kinetic studies, we revealed a role for EL in determining APOB-containing particle composition and clearance upon ANGPTL3 inhibition. We found that EL modifications on VLDL were necessary for LDLR-independent particle removal at the juncture between VLDL remnants and their further processing to become LDL. In the absence of both EL and LDLR, ANGPTL3 inhibition led to generation of partially processed remnants, which blunted their clearance and led to accumulation of TG-depleted cholesterol- and phospholipid-rich LDL.

Such a scenario can be explained by EL-dependent VLDL processing in the circulation but is more difficult to reconcile with reduced hepatic VLDL output. In fact, ANGPTL3 inhibition did not affect the hepatic transcriptome, VLDL assembly, and liver lipid content (supplemental Figure S5). In this regard, our findings differ from previous studies reporting lower VLDL-TG secretion (24) and reduced liver fat (10) upon ANGPTL3 inhibition. Prior evaluation of VLDL-TG secretion depended on lipolysis inhibitor Triton WR-1339 (24). We speculate that the observed VLDL-TG reduction may be due to rapid unrestrained LPL activity toward nascent VLDL upon ANGPTL3 inhibition and/or incomplete LPL inhibition by Triton WR-1339. If ANGPTL3 inhibition indeed led to secretion of smaller VLDL with lower TG content, our data from EL-deficient mice (where lipolysis led to accumulation of TG-depleted remnants) suggest that TG reduction on its own may have little impact on the clearance of these particles. That said, it is still feasible that the combination of altered VLDL-TG secretion and EL-driven catabolism of modified particles ultimately promotes VLDL remnant clearance and thus LDL-C reduction. Subsequent studies in *Lpl* conditional KO mice, or even *Lipg/Lpl* double KO mice, should resolve these discrepancies, as the absence of lipolysis will allow us to better determine the lipid composition of nascent VLDL upon ANGPTL3 blockade.

Regarding liver fat, ASO-mediated *Angptl3* inhibition was found to reduce liver TG content (10), whereas evinacumab had no such effects (supplemental Fig. S5C). While this key difference may be rooted in the mechanism of pharmacological inhibition (modulating hepatic gene expression vs. protein blockade in the circulation), it is noteworthy that *Angptl3*^−/−^ mice have similar liver TG content as WT mice (40). Moreover, in contrast to our current and prior studies (24), Musunuru et al. (6) reported decreased VLDL-APOB production rates in humans with *ANGPTL3* LOF variants. However, these findings were limited to two homozygous and three heterozygous *ANGPTL3* LOF carriers. A recent NMR study on a larger number of *ANGPTL3* LOF carriers found that among APOB-lipoproteins, the greatest cholesterol-lowering effect was in the VLDL remnant fraction (56), consistent with our findings in mice. Notably, there are several differences in lipid metabolism between mice and humans. Mice are genetically deficient for ChoE transfer protein (CETP) and, hence, have low LDL-C and carry the majority of cholesterol on HDL (57). Mice also secrete VLDL containing either APOB-48 or APOB-100, while human VLDL only contains APOB-100 (58). Thus, studies in larger populations of humans will be necessary to evaluate whether our findings in mice are consistent with humans, or if altered VLDL secretion and catabolism both contribute to the LDL-C-lowering effect. Eventually, only studies in homozygous FH patients treated with ANGPTL3 inhibitor may be able to provide definitive answers, given that the EL-mediated pathway we discovered in mice only becomes critical in the absence of functional LDLR.

Nonetheless, evinacumab’s action on VLDL remnants represents a mechanism that is distinct from existing lipid-lowering treatments, including statins and PCSK9, MTP, and APOB inhibitors (54). Consistent with this notion, a recent publication revealed that triple combination treatments of statins and antibodies targeting PCSK9 and ANGPTL3 yielded additive effects on cholesterol and atherosclerosis reductions in mice (59). ANGPTL3 inhibition even operates differently than APOC3 or ANGPTL4 blockade, which also activate LPL and promote TG hydrolysis but have only minor effects on cholesterol reduction (60–62). These findings illustrate that increased rates of hydrolysis are not invariably linked to greater hepatic particle clearance. While individuals with heterozygous loss of APOC3 exhibit increased conversion of VLDL to LDL, no concomitant increase in VLDL remnant clearance kinetics was seen (63). We observed similar effects in EL/LDLR double KO mice, in which the absence of EL impaired VLDL remnant clearance typically seen with ANGPTL3 inhibition and promoted its conversion to LDL. Indeed, the key distinction between APOC3 and ANGPTL3 appears to be their differential ability to modulate EL: unlike for ANGPTL3, APOC3 has not been reported to affect EL activity. Thus, ANGPTL3 deficiency represents a unique scenario where derepression of both LPL and EL drives VLDL remodeling and its LDLR-independent clearance, thereby restricting LDL production.

In regard to clearance, our studies have unmasked multiple layers of redundancy. ANGPTL3 inhibition effectively lowered LDL-C in *Lipg*^−/−^ (single KO) mice, likely due to the presence of LDLR and its ability to bind VLDL remnants (1, 3). Without LDLR, EL activity becomes critical, yet clearance of EL-modified VLDL may involve multiple redundant receptors (47–51). Deletion of LRP1 and SDC1 were previously shown to be dispensable for evinacumab’s LDL-C-lowering effect (24). While in the current study our combinatorial approach to blunt hepatic LDLR/SDC1/LRP1/SR-B1 activities did not abolish LDL-C reduction upon ANGPTL3 inhibition, it is feasible that their extrahepatic expression may contribute to evinacumab’s effect as well. Future tissue uptake studies with radiolabeled VLDL will help to pinpoint the potential clearance receptor(s). Coupling these studies with VLDL, dually labeled with TG and cholesteryl (or retinyl) ester tracers will reveal further insights into the relative kinetics of TG lipolysis versus remnant uptake.

How then does EL activation lead to VLDL clearance? Given that EL functions as a phospholipase, its impact on cholesterol is thought to be secondary to lipoprotein remodeling (20). We surmise that the reduction of surface phospholipids may expose additional APOB domains, which facilitate remnant receptor interactions and lipoprotein uptake. Conversely, the lack of EL may generate phospholipid-enriched remnants of discoid or irregular shape and size, which potentially prevents efficient interaction with hepatocytes. EL could also change VLDL apolipoprotein content and alter plasma clearance rates independently of APOE (24) in this way. Electron microscopy and proteomic approaches will shed light on the role of EL on APOB lipoprotein structure and composition in future studies. Besides EL, HL is also known to hydrolyze phospholipids (20, 64); yet prior studies have shown that ANGPTL3 does not regulate HL activity (11, 40). In vitro data suggest that even LPL has low phospholipase activity (64–66); however, vascular lipases seem to have broader substrate specificity in vitro, as EL was also shown to exhibit appreciable, albeit weak, TG-lipase activity in assays with purified enzyme (64). Our in vivo studies show that LPL activation had no substantial effect on phospholipids and particle clearance in EL/LDLR double KO mice (Fig. 1E). Thus, neither HL nor LPL appear to be able to compensate for EL derepression when LDLR is missing, potentially due to competition between high levels of HDL and APOB-lipoproteins for lipolytic enzymes. Finally, it should be noted that although EL’s enzymatic activity led to substantial remodeling of APOB-lipoproteins, its nonenzymatic “bridging” function may also facilitate remnant clearance through uptake receptors (49).

In summary, we have uncovered a novel mechanism that lowers LDL-C by ANGPTL3 inactivation. Through derepression of EL, ANGPTL3 inhibition promotes VLDL processing and clearance. This reduces LDL production and bypasses the requirement for LDLR (see schematic in Fig. 6). While EL appears to be dispensable for LDL-C regulation in normal individuals, its effect on VLDL remodeling and clearance becomes critical without LDLR. Together, these studies advance our understanding of lipoprotein metabolism and provide important insights into the mechanism by which ANGPTL3 inhibition lowers LDL-C in patients lacking functional LDLR.

### Data availability

All data associated with this study are present in the paper or the supplemental Materials, and all figures except Fig. 6 (schematic) have associated raw data. Any materials that can be shared will be released via a material transfer agreement. The accession number for the sequencing data reported in this paper is NCBI Gene Expression Omnibus: GSE143726.

## Supplementary Material

Supplemental Data
