# Supplementary material for: Angiopoietin-like protein 3 governs LDL-cholesterol levels through endothelial lipase-dependent VLDL clearance
Source: J Lipid Res. 2020 Jul 9;61(9):1271–86. doi: 10.1194/jlr.RA120000888 (PMC7469887; doi:10.1194/jlr.RA120000888)
Supplement: Supplemental Data [file supp_61_9_1271__index.html]

Angiopoietin-like protein 3 (ANGPTL3) governs LDL-cholesterol levels through endothelial lipase-dependent VLDL clearance — ANGPTL3 governs LDL-C levels by regulating VLDL catabolism — Angiopoietin-like protein 3 governs LDL-cholesterol levels through endothelial lipase-dependent VLDL clearance — Supplemental Data 

# Angiopoietin-like protein 3 governs LDL-cholesterol levels through endothelial lipase-dependent VLDL clearance

## Supplemental Data

- Supplemental Figures - Supplemental Figures S1-S7 and figure legends
- Supplemental Table S1 - Human genetic associations with lipid levels
- Supplemental Table S2 - HDL lipidomics
- Supplemental Table S3 - LDL lipidomics
- Supplemental Table S4 - VLDL lipidomics
- Supplemental Table S5 - Liver transcriptome analysis
- VLDL APOB Western Blot - full scan of WB
- LDL APOB Western Blot - full scan of WB
