# Supplementary material for: Angiopoietin-like protein 3 governs LDL-cholesterol levels through endothelial lipase-dependent VLDL clearance
Source: J Lipid Res. 2020 Jul 9;61(9):1271–86. doi: 10.1194/jlr.RA120000888 (PMC7469887; doi:10.1194/jlr.RA120000888)
Supplement: Supplemental Data [file supp_RA120000888_160666_2_supp_558046_qcsddh.pdf]

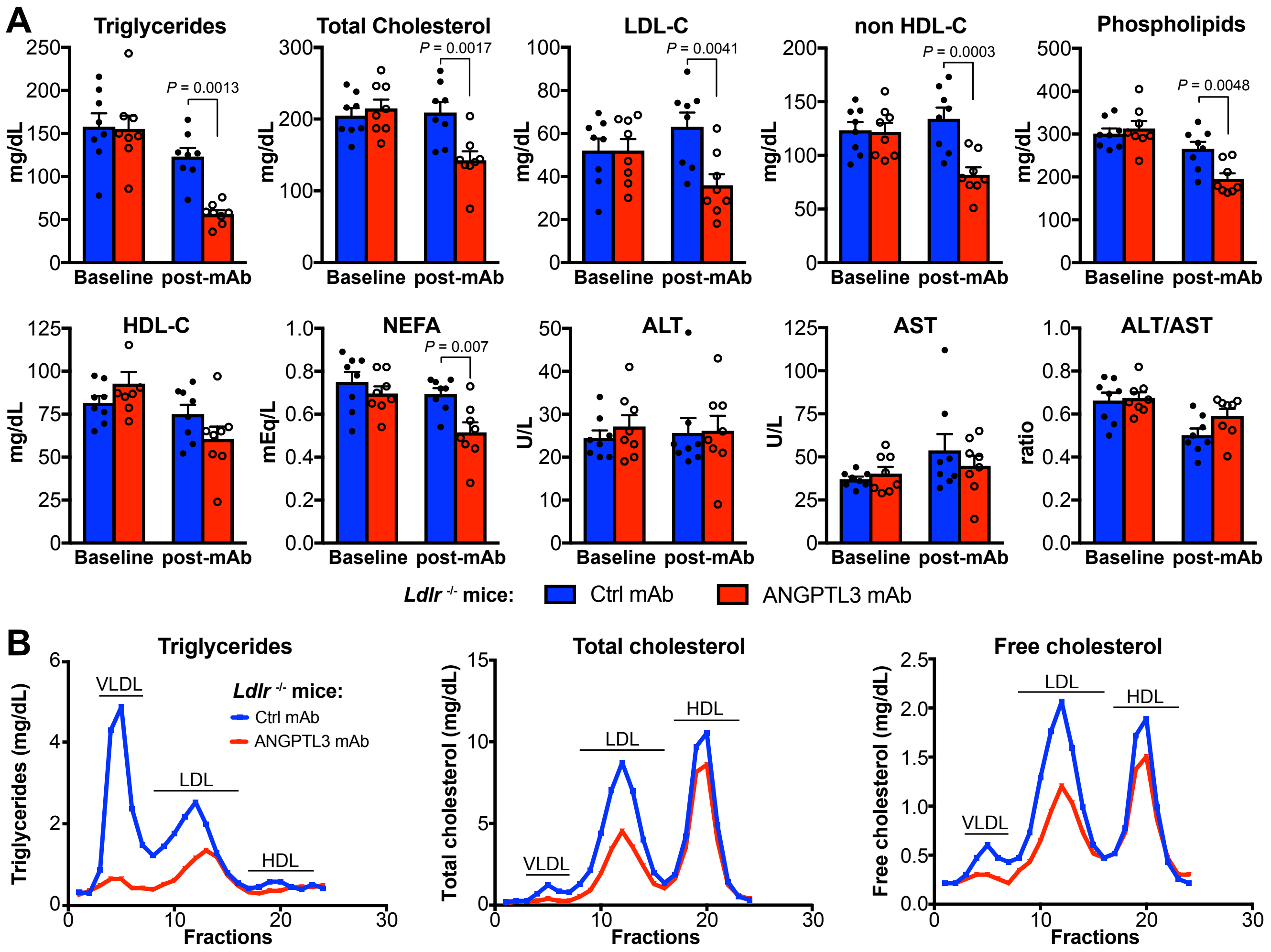

Supplemental Fig. S1. ANGPTL3 inhibition lowers serum lipids independently of LDLR. A: Non-fasted serum lipids and liver enzyme levels 4 days before (baseline) first, and 3 days after second mAb administration. Mean ± s.e.m. are shown (n = 8 mice/group). P-values are from two-way ANOVA with Sidak correction posttest. B: Serum lipid distribution in *Ldlr*$^{-/-}$ mice showing ANGPTL3 mAb lowers lipids across all lipoproteins. Pooled serum (n = 6 mice in Ctrl mAb group, 5 in ANGPTL3 mAb group) was fractionated by FPLC, and cholesterol and TG levels were measured enzymatically in each fraction. All mice were on chow diet.

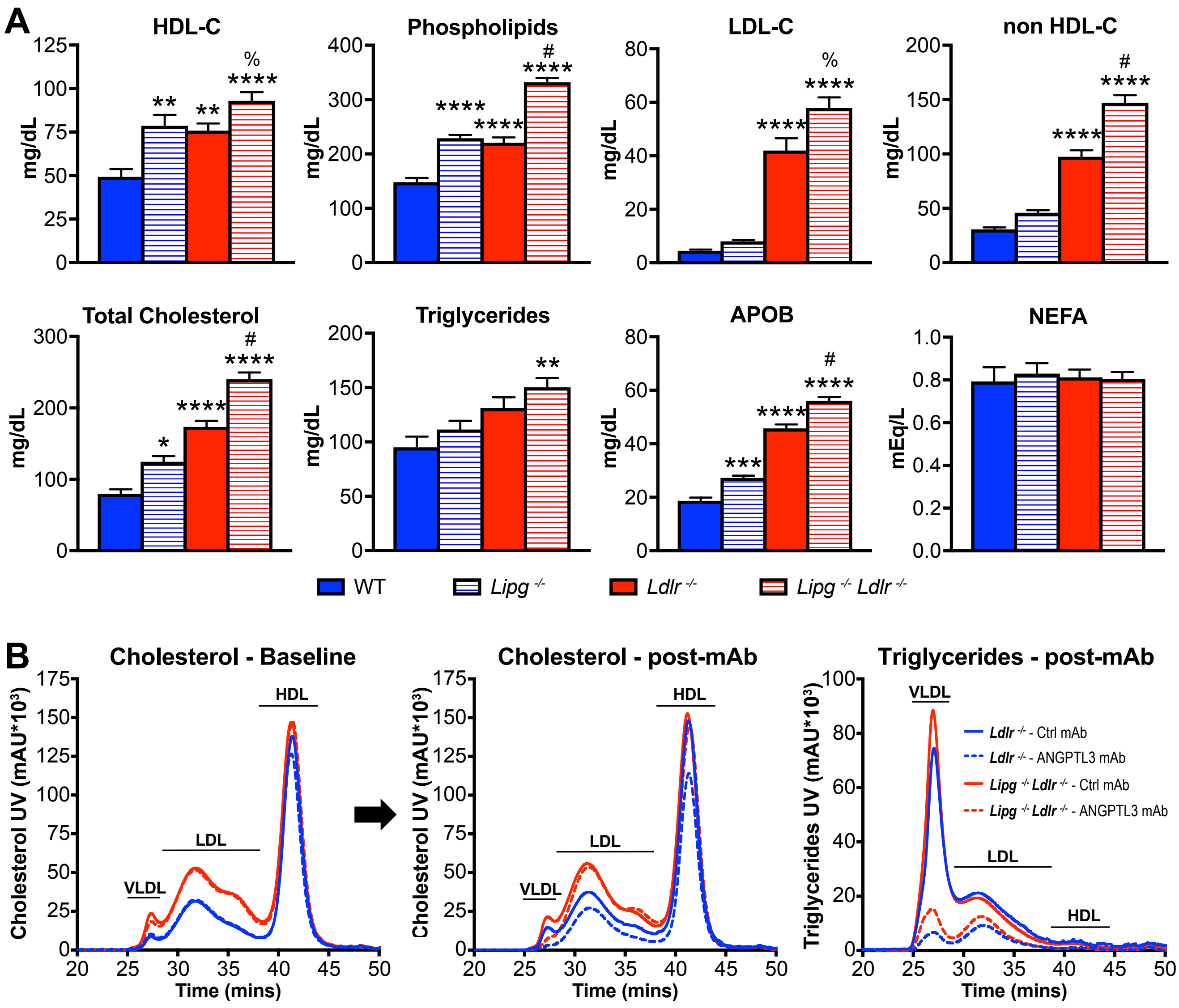

Supplemental Fig. S2. Endothelial lipase contributes to APOB-lipoprotein metabolism. A: Non-fasted serum lipids of WT (*n* = 10), *Lipg*$^{-/-}$ (*n* = 12), *Ldlr*$^{-/-}$ (*n* = 14 for phospholipids and APOB, 23 for all other lipids) and *Lipg*$^{-/-}$*Ldlr*$^{-/-}$ (*n* = 18 for phospholipids and APOB, 24 for all other lipids) mice on chow diet. Mean ± s.e.m. are shown. P-values from one-way ANOVA with Tukey correction posttest: **P<0.01, ***P<0.001, ****P<0.0001 relative to WT. $^{\%}$P<0.05 relative to *Ldlr*$^{-/-}$. $^{\#}$P<0.0001 relative to *Ldlr*$^{-/-}$. B: HPLC analysis of serum lipid distribution in *Ldlr*$^{-/-}$ and *Lipg*$^{-/-}$*Ldlr*$^{-/-}$ mice before (baseline) and 7 days after mAb-administration. All mice were on chow diet.

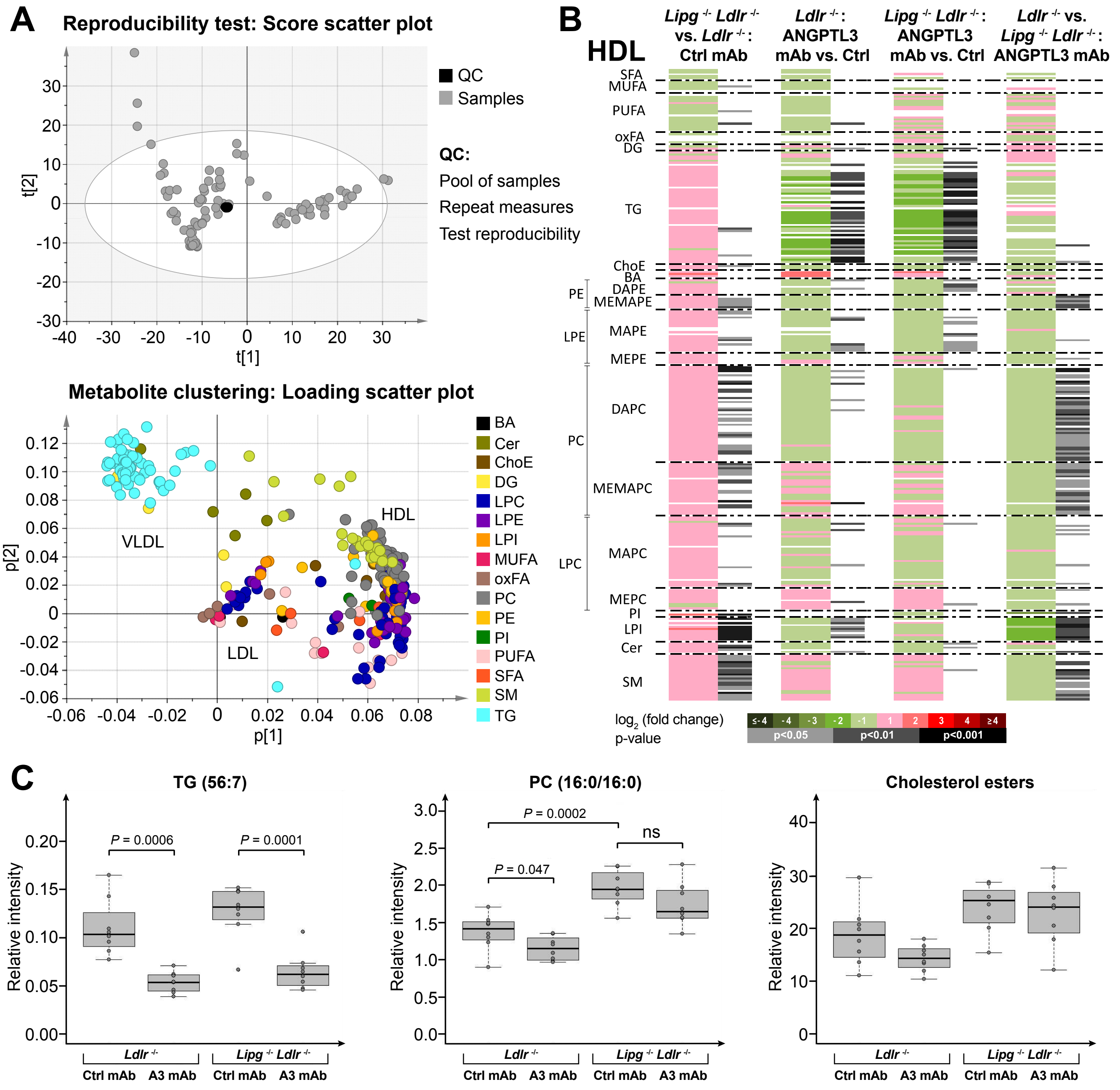

Supplemental Fig. S3. Lipidomics reveals EL's role in HDL remodeling upon ANGPTL3 inhibition. A: (top) Score scatter plot (unsupervised principal component analysis (PCA) model) of the metabolic profiles of all samples and QC. Model diagnostics (A=6; R2X=0.877; Q2X=0.826). (bottom) Metabolite loadings plot of the PCA model including VLDL, LDL and HDL lipoproteins. Model diagnostics (A=6; R2X=0.872; Q2X=0.813). B: Heatmap representing individual metabolites, denoted on the left, obtained for the comparisons performed in HDL samples (*n* = 8 mice/group). Heatmap color codes for $\log_2$ (fold-change) and unpaired two-tailed Student's t-test p-values are

indicated at the bottom. C: Boxplots (defined in Methods) of HDL-lipids upon ANGPTL3 mAb or isotype control. P-values are from unpaired two-tailed Student's t-test. All mice were on chow diet. See also lipidomics data including list of metabolite abbreviations in supplemental Table S2.

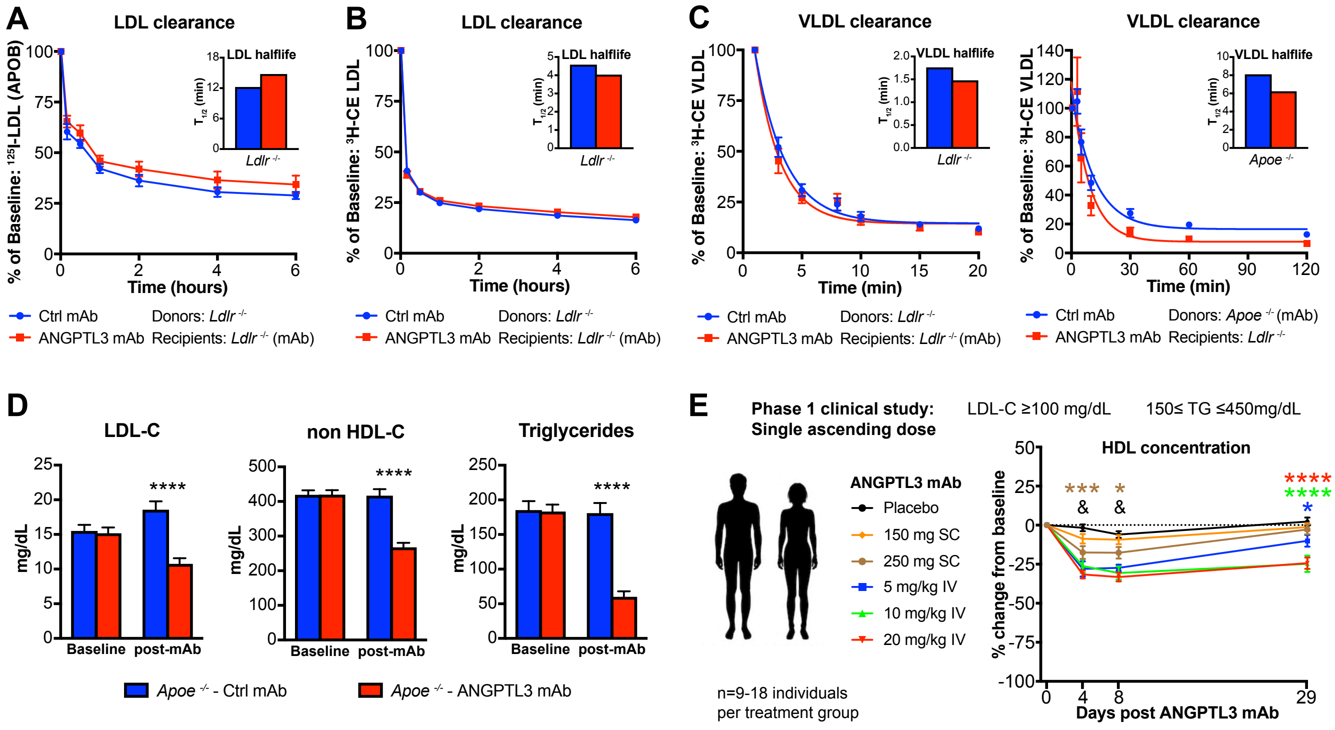

Supplemental Fig. S4. Lipoprotein clearance kinetics upon ANGPTL3 inhibition. A-B: Plasma clearance kinetics of [^{125}I]-labeled LDL (A), or [^{3}H]-CE-labeled LDL (B), isolated from and injected into *Ldlr*$^{-/-}$ mice after 2 weekly injections of ANGPTL3 mAb or isotype control (n = 8 mice/group). APOB (A) was precipitated from plasma using isopropanol and the radioactivity was quantitated in a scintillation counter. The percentage of injected label remaining at each timepoint was determined using the value obtained at 1min as starting point. Mean ± s.e.m. are shown for each timepoint. The half-life was calculated from the decay curve of APOB activity plotted against time. C: (left) Plasma clearance kinetics of [^{3}H]-CE-labeled VLDL, isolated from and injected into *Ldlr*$^{-/-}$ mice after 2 weekly injections of ANGPTL3 mAb or control mAb (n = 8 mice in Ctrl mAb group, 6 in ANGPTL3 mAb group). CE, cholesteryl ether. (right), Plasma clearance kinetics of [^{3}H]-CE-labeled VLDL, isolated from *Apoe*$^{-/-}$ mice after 2 weekly injections of ANGPTL3 mAb or control mAb, and injected into *Ldlr*$^{-/-}$ mice (n = 10 mice in Ctrl mAb group, 9 in ANGPTL3 mAb group). For both studies, mean ± s.e.m. are shown for each timepoint. The percentage of injected label remaining was determined using the value obtained at 0.5 or 1min as starting point. The half-life was calculated from the decay curve of [^{3}H]-CE activity plotted against time. In kinetic studies, all mice were on chow diet. D: Non-fasted serum lipids of *Apoe*$^{-/-}$ mice on chow diet before (baseline, Day -3) and 3 days after the second once/weekly ANGPTL3 or control mAb-dose (10mg/kg). Mean ± s.e.m. are shown (n = 32 mice in each group. P-values are from

two-way ANOVA with Sidak correction posttest. ****P<0.0001. Lipoproteins purified from these mice were used in kinetic studies in Fig. 4B. E: Nuclear magnetic resonance (NMR) analysis of human HDL following ANGPTL3 inhibition. Hyperlipidemic individuals were administered ANGPTL3 mAb or control mAb (Phase 1 single ascending dose clinical study) (28), and serum was collected at the indicated timepoints. Mean ± s.e.m. are shown for each timepoint (*n* = 18 in placebo; 10 in 5mg/kg IV; 9 in 10mg/kg IV; 11 in 20mg/kg IV; 12 in 150mg SC and 9 in 250mg SC groups). Human HDL concentration (right) analysis by NMR. P-values from two-way ANOVA with Tukey correction posttest: *P<0.05, ***P<0.001, ****P<0.0001 relative to placebo (color of asterisks indicate relevant group); $^{\&}$P<0.0001 regarding all IV-administered ANGPTL3 mAb groups relative to placebo.

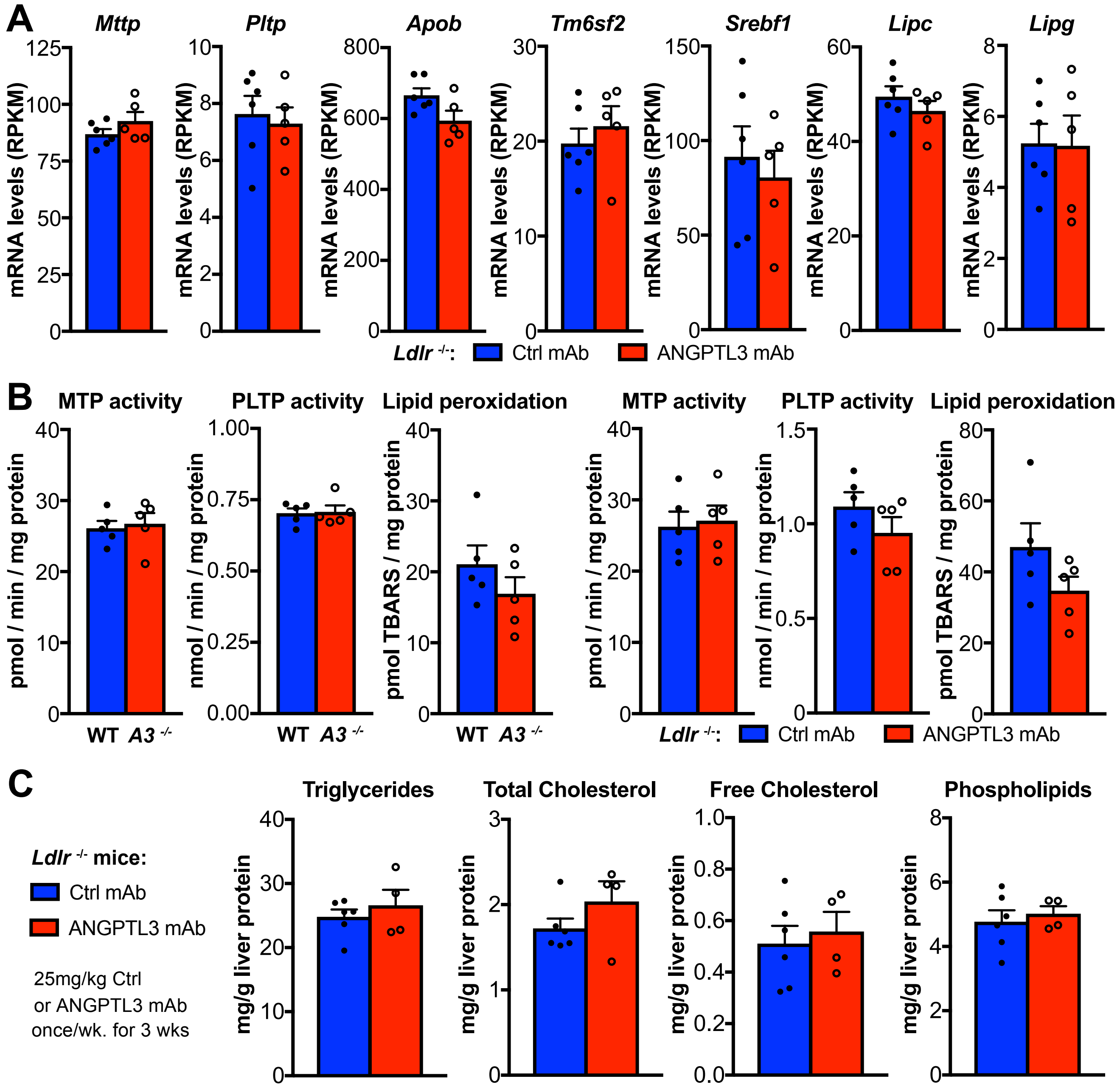

Supplemental Fig. S5. ANGPTL3 inhibition does not impair hepatic lipid homeostasis. A: Liver transcriptome analysis in *Ldlr*$^{-/-}$ mice: mRNA levels of EL (*Lipg*) and hepatic lipase (*Lipc*), and genes involved in hepatic VLDL assembly after multi-dose ANGPTL3 mAb or isotype control. Mean ± s.e.m. are shown (*n* = 6 mice in Ctrl mAb group, 5 in ANGPTL3 mAb group). B: Enzymatic activity assays (MTP, PLTP) in liver homogenates of *Angptl3*$^{-/-}$ (*A3*$^{-/-}$) mice, or *Ldlr*$^{-/-}$ mice treated with ANGPTL3 mAb. TBARS are a readout for lipid peroxidation. Mean ± s.e.m. are shown (*n* = 5 mice/group). C: Hepatic lipid content following multi-dose ANGPTL3 mAb or control mAb

administration. Mean ± s.e.m. are shown (*n* = 6 mice in Ctrl mAb group, 4 in ANGPTL3 mAb group). All mice were on chow diet. See also supplemental Table S5 for complete transcriptome data.

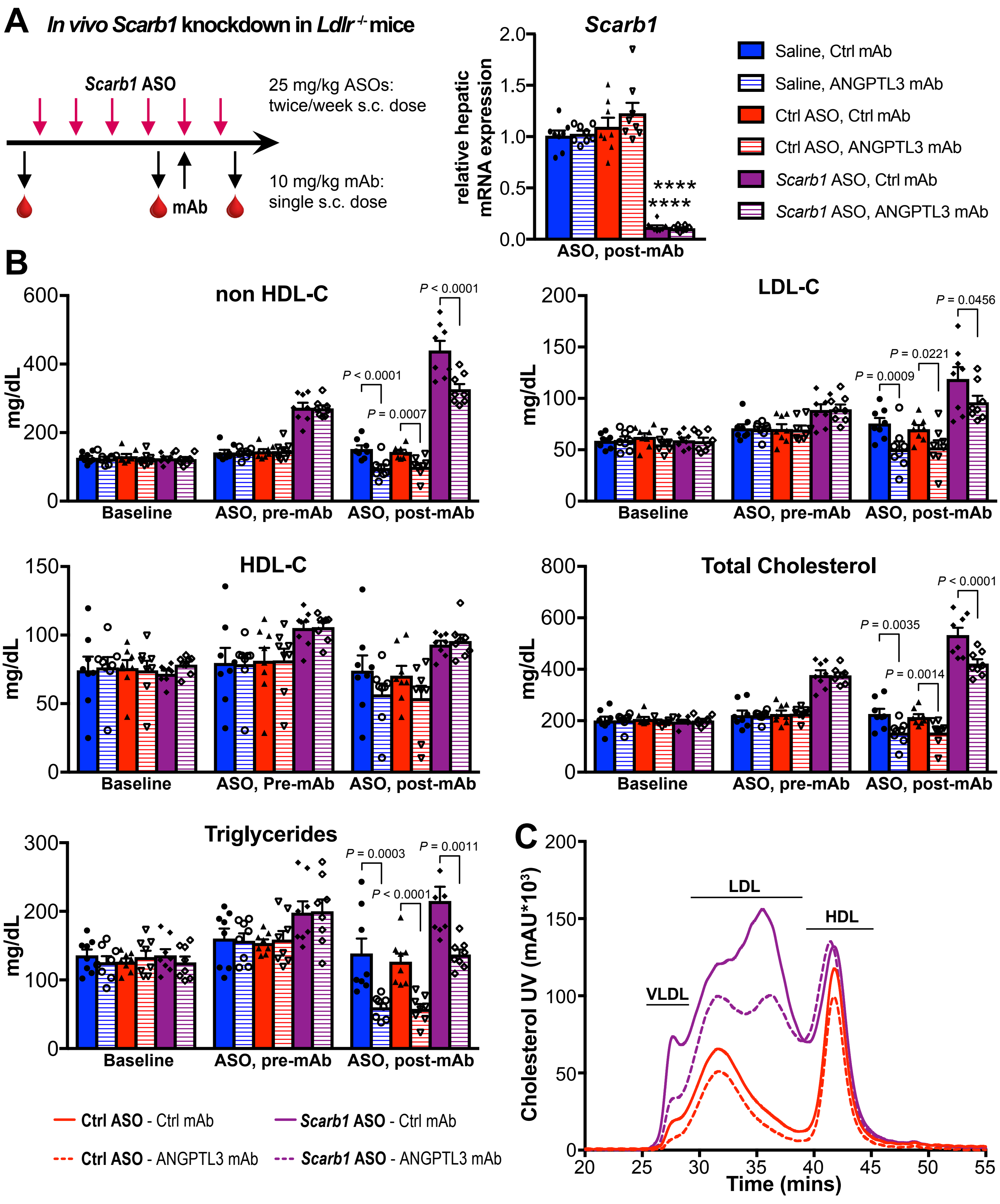
A In vivo Scarb1 knockdown in Ldlr-/- mice
Scarb1 ASO
25 mg/kg ASOs: twice/week s.c. dose
mAb
10 mg/kg mAb: single s.c. dose
Scarb1
relative hepatic mRNA expression
ASO, post-mAb
Saline, Ctrl mAb
Saline, ANGPTL3 mAb
Ctrl ASO, Ctrl mAb
Ctrl ASO, ANGPTL3 mAb
Scarb1 ASO, Ctrl mAb
Scarb1 ASO, ANGPTL3 mAb
B
non HDL-C
LDL-C
HDL-C
Total Cholesterol
Triglycerides
mg/dL
Baseline
ASO, pre-mAb
ASO, post-mAb
P < 0.0001
P = 0.0007
P = 0.0456
P = 0.0221
P = 0.0009
P = 0.0035
P = 0.0014
P = 0.0003
P = 0.0011
Ctrl ASO - Ctrl mAb
Ctrl ASO - ANGPTL3 mAb
Scarb1 ASO - Ctrl mAb
Scarb1 ASO - ANGPTL3 mAb
C
Cholesterol UV (mAU*10³)
VLDL
LDL
HDL
Time (mins)

Supplemental Fig. S6. SR-B1 is dispensable for LDL-C reduction upon ANGPTL3 inhibition. A: Experimental design and hepatic knockdown efficiency upon antisense oligonucleotide (ASO)-mediated knockdown of *Scarb1* in *Ldlr*$^{-/-}$ mice on chow diet. Serum was collected to determine lipid levels of *Ldlr*$^{-/-}$ mice at baseline. *Scarb1* or control ASO was administered by twice-weekly s.c. injections at 25mg/kg, for a total 6 doses. ANGPTL3 or control mAb (10mg/kg) were administerd after the first 4 doses of ASO (ASO, pre-mAb). Serum was collected 7 days after mAb administration (ASO, post mAb). Livers were harvested to determine *Scarb1* expression 7 days after mAb-administration. P-values are from one-way ANOVA with Sidak correction posttest: ****P<0.0001. B: Non-fasted serum lipids of saline, *Ctrl* ASO and *Scarb1* ASO-treated *Ldlr*$^{-/-}$ mice on chow diet. P-values are from two-way ANOVA with Sidak correction posttest. C: HPLC analysis shows serum lipid distribution of chow-fed mice. Mean ± s.e.m. are shown (*n* = 8 mice/group).

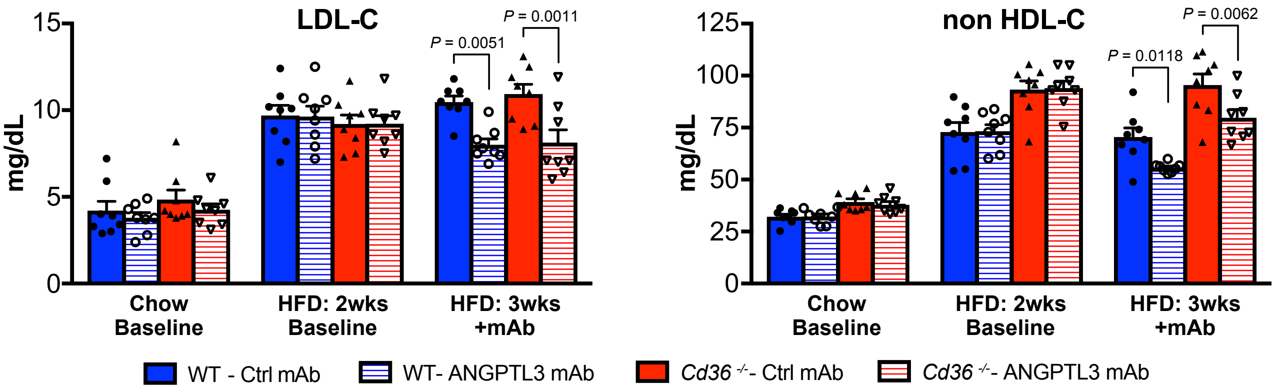

Supplemental Fig. S7. ANGPTL3 inhibition lowers LDL-C independently of CD36. Non-fasted serum lipids of WT and *Cd36*$^{-/-}$ mice on chow (baseline), after 2 weeks on high-fat diet (HFD baseline), and upon mAb-administration while on HFD. Serum was collected 6 days after ANGPTL3 or control mAb-dose (25mg/kg). Mean ± s.e.m. are shown (n = 8 mice/group). P-values are from two-way ANOVA with Tukey correction posttest.

Supplemental Tables

Supplemental Table S1. Human genetic associations of *LIPG* variants with plasma lipid levels.

Supplemental Table S2. HDL lipidomic analysis of *Ldlr*$^{-/-}$ and *Lipg*$^{-/-}$*Ldlr*$^{-/-}$ mice.

Supplemental Table S3. LDL lipidomic analysis of *Ldlr*$^{-/-}$ and *Lipg*$^{-/-}$*Ldlr*$^{-/-}$ mice.

Supplemental Table S4. VLDL lipidomic analysis of *Ldlr*$^{-/-}$ and *Lipg*$^{-/-}$*Ldlr*$^{-/-}$ mice.

Supplemental Table S5. Liver transcriptome analysis of *Ldlr*$^{-/-}$ mice treated with multi-dose ANGPTL3 mAb or isotype control mAb.
