# Supplementary figures and images for: Angiopoietin-like protein 3 governs LDL-cholesterol levels through endothelial lipase-dependent VLDL clearance
Source: J Lipid Res. 2020 Jul 9;61(9):1271–86. doi: 10.1194/jlr.RA120000888 (PMC7469887; doi:10.1194/jlr.RA120000888)

# VLDL fractions

anti-APOB (Millipore, AB742), 1:1000

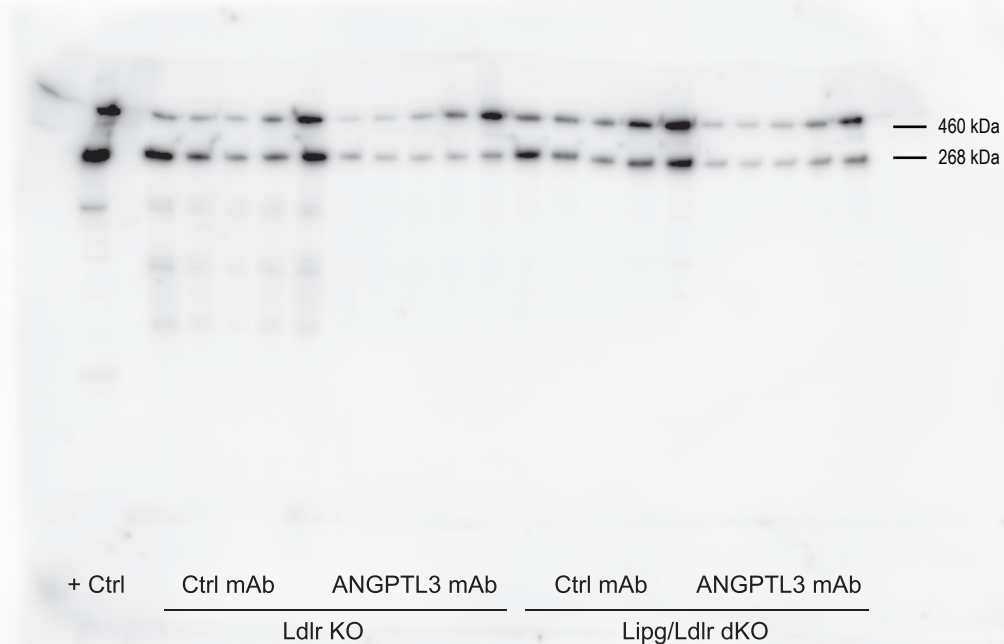

Supplement: Supplemental Data [file supp_RA120000888_160666_2_supp_561070_qd3wy3.pdf]
